# The induced-fit and catalytic mechanisms of human G6PC1

**DOI:** 10.1038/s41421-025-00814-z

**Published:** 2025-07-15

**Authors:** Qihao Chen, Yuhang Wang, Renjie Li, Qinru Bai, Yan Zhao

**Affiliations:** 1 Key Laboratory of Biomacromolecules (CAS), National Laboratory of Biomacromolecules, CAS Center for Excellence in Biomacromolecules, Institute of Biophysics, Chinese Academy of Sciences, Beijing, China; 2 College of Life Sciences, University of Chinese Academy of Sciences, Beijing, China

**Keywords:** Molecular biology, Cryoelectron microscopy

## Abstract

Human glucose-6-phosphatase catalytic subunit 1 (hG6PC1) is a key enzyme in glucose metabolism, governing the final common step of gluconeogenesis and glycogenolysis, and directly regulating energy homeostasis. Aberrant mutations in G6PC1 directly cause glycogen storage disease type 1a, which is characterized by chronic hypoglycemia and glycogen accumulation. Additionally, abnormal G6PC1 function leads to increased fasting blood glucose. Consequently, it is a critical target for treating glucose metabolism disorders. In this study, we determine the cryo-EM structures of G6PC1 in both the partially open and fully open states, in either the apo form or in complex with the substrates G6P or F6P and the product phosphate. These structures offer distinct insights into the mechanism of hydrolysis and induced-fit, providing a structural foundation for the diagnostic analysis of disease-causing mutations in G6PC1. Moreover, we propose a potential mechanism by which phosphatidylserine regulates G6PC1 activity, providing a novel perspective on its role and implications.

## Introduction

In energy metabolism, glucose serves as the most essential energy source for all organisms^1^. In particular, the brain relies almost exclusively on glucose as its sole fuel owing to the selective permeability of the blood‒brain barrier, necessitating a constant supply of glucose via the bloodstream^2,3^. Glucose homeostasis is tightly regulated through several pathways, including glycogenesis, glycogenolysis, glycolysis, and gluconeogenesis^4^. Among these, glycogenolysis and gluconeogenesis are responsible for synthesizing glucose from glycogen and pyruvate, respectively, which are crucial for maintaining glucose levels during fasting, particularly in tissues, such as the brain, that rely almost exclusively on glucose^5^. Disruptions in these processes directly lead to imbalances in blood sugar levels, resulting in conditions such as hypoglycemia^4^ or diabetes^6^. The accumulation of metabolic byproducts can cause severe complications, such as lactic acidosis^7^ and lipid metabolism disorders^8^, posing serious risks to patients’ lives. Human glucose-6-phosphatase (G6Pase) is the rate-limiting enzyme in both glycogenolysis and gluconeogenesis, controlling the common final process of these pathways. G6PC1 catalyzes the conversion of G6P to free glucose, which is then transported to various tissues through the bloodstream, supporting essential physiological functions^9^.

Extensive studies have shown that aberrant mutations in G6PC1, which reduce or eliminate catalytic activity, cause glycogen storage disease type 1a (GSD-1a; MIM #232200)^10–12^. It accounts for 80% of all GSD cases, with an annual incidence of approximately 1 in 100,000^13^. Without treatment, GSD-1a can lead to chronic and complex complications, including growth retardation, hepatosplenomegaly, nephromegaly, hyperlactatemia, hyperuricemia, and hyperlipidemia^14^. Although the consumption of uncooked cornstarch is used to manage GSD-1a, the risk of severe hypoglycemia and chronic complications persists^15^. Currently, gene therapies targeting G6PC1 have shown the potential to correct hypoglycemia and prevent other metabolic disorders in GSD-1a animal models^16–18^ and in a clinical trial (ClinicalTrials.gov Identifier: NCT03517085). A *G6PC1* mRNA delivery platform has also demonstrated favorable efficacy and safety in GSD-1a preclinical studies^19^ and is now being further evaluated in a clinical trial (ClinicalTrials.gov Identifier: NCT05095727). Additionally, increased G6PC1 expression in diabetic mice suggests a link between elevated G6PC1 activity and disrupted glucose metabolism in diabetes^20,21^. In diabetes patients, insulin resistance probably results in G6PC expression elevation, leading to the continuous hepatic glucose production^22,23^. Endogenous fructose is generated in the liver through the polyol pathway^24^. The activity of this pathway increases significantly during hyperglycemia^25^, and excess endogenous fructose can lead to various metabolic disturbances, including increased body weight, visceral obesity, fatty liver, elevated insulin levels, and hyperleptinemia. Recent research suggests that G6PC1 may catalyze the conversion of fructose-6-phosphate to fructose^9^. Furthermore, elevated fructose levels are linked to the upregulation of G6PC1 activity^26^. Thus, we propose that G6PC1 may contribute to the metabolic disorders associated with diabetes and obesity, potentially influenced by fructose metabolism. The key role of G6PC1 in maintaining glucose homeostasis makes it a focal point for developing new treatments for glucose metabolic disorders, particularly those involving impaired glucose regulation.

G6PC1 belongs to the type II phosphatidic acid phosphatase (PAP2) family and is selectively expressed in the endoplasmic reticulum (ER) membrane within the liver, kidneys, and small intestine^9,27^. Over the past twenty years, several structural studies on prokaryotic members of the PAP2 family have revealed the hydrolysis mechanism of amphipathic phospholipid-like substrates and the binding pattern of hydrolyzed products^27–31^. However, the absence of structures in different conformations induced by substrate binding impedes the knowledge of the detailed mechanism of the induced-fit model in the PAP2 family. In addition, as a completely different phosphocarbohydrate molecule from phospholipid-like molecules, the unique recognition pattern and specific hydrolysis mechanism of G6P are still unresolved. Given the significant differences in sequence, it is likely that eukaryotic G6PC1, which serves as the G6P hydrolysis protein, has distinct folding patterns. Therefore, how G6PC1, which is structurally completely different from other members of the PAP2 family, results in similar dephosphorylation of a completely different hydrophilic G6P substrate remains ambiguous. Although several studies have revealed the partial catalytic mechanism of G6PC1, systematic and detailed information remains unclear^32–34^. In addition, the effects of substrate binding-induced conformational changes on G6PC1 activity have not been investigated. Therefore, precise observation of the specific details of the G6PC1-catalyzed process based on its structure is crucial for addressing these issues.

To explore substrate recognition and the enzymatic hydrolysis mechanism of G6PC1, as well as further elucidate the impact of disease-causing mutations, we determined distinct conformational states of G6PC1 using cryo-electron microscopy (cryo-EM) in its apo state and in complex with substrates and products. These structures depict a distinct landscape of both the enzymatic reaction and the induced-fit model of G6PC1 and reveal the structural and mechanistic basis of the catalytic dysfunction caused by GSD-1a mutations, providing essential insights for the design of small molecules targeting G6PC1 activity.

## Results

### Overall structure of G6PC1

To elucidate the specific mechanism by which G6PC1 recognizes and hydrolyzes G6P, we expressed full-length wild-type (WT) G6PC1 tagged with GFP in HEK293F cells. G6PC1 purified using lauryl maltose neopentyl glycol (LMNG) detergent exhibited significant hydrolytic activity and a Michaelis constant (*K*_m_) of ~2 mM (Supplementary Fig. S1a). This *K*_m_ value was consistent with those reported in the previous study^35^. Consequently, we purified G6PC1 via the detergent LMNG and reconstituted it into nanodiscs. After removing GFP and twin-strep tags by enzyme digestion, the untagged G6PC1 was used for cryo-EM structural analysis (Supplementary Fig. S1d–f). We successfully determined the structures of G6PC1 bound to the product phosphate, the substrates glucose-6-phosphate (G6P) or fructose-6-phosphate (F6P) at resolutions of 3.1 Å, 2.9 Å and 3.3 Å, respectively (Supplementary Figs. S2, S4, S5). Additionally, to explore the changes during substrate binding and product release in G6PC1, we determined its cryo-EM structure in the apo state at a resolution of 3.4 Å (Supplementary Fig. S3). These structures are referred to as G6PC1^Pi^, G6PC1^G6P^, G6PC1^F6P^, and G6PC1^APO^, respectively.

**Fig. 1:**
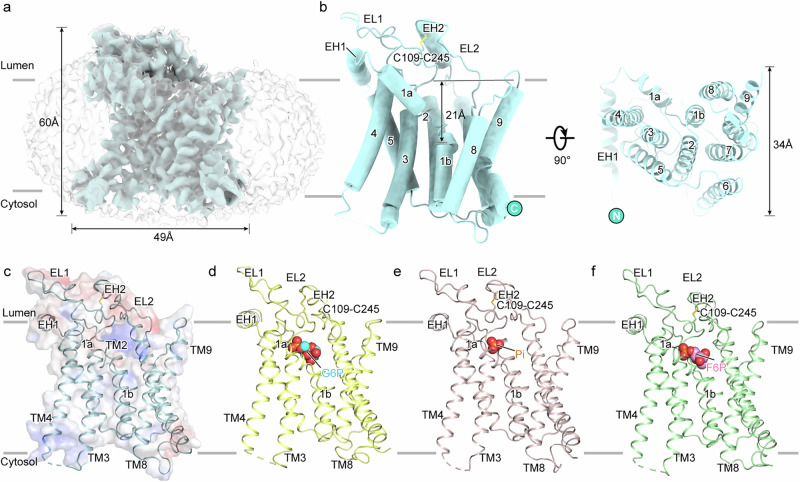
Architecture of the hG6PC1. **a** Cryo-EM map of hG6PC1 in nanodiscs observed from a view parallel to the membrane. The membrane plane is indicated as two grey lines and the directions are labeled. The structure of hG6PC1 exhibits dimensions of approximately 49 Å in length, 34 Å in width, and 60 Å in height. **b** Overall structure of hG6PC1 in side view (left) and top-down view (right). N- and C-termini are labeled with blue circles. EH1–2, EL1–2 and TM1–9 are indicated. The width of hG6PC1 is indicated. The distance between the N-terminus of TM1 and the membrane in the luminal side is labeled. **c**–**f** Overall structures of hG6PC1 in its apo state (**c**) and in complex with G6P (**d**) or phosphate (**e**) or F6P (**f**). Pi phosphate. These four structures are shown as cartoon and colored light blue, pale yellow, light pink, and pale green, respectively. The ligands are depicted as spheres and labeled. The surface electrostatic potential of G6PC^APO^ is calculated with APBS and shown in **c**.

G6PC1 contains a transmembrane domain (TMD) composed of nine transmembrane helices and an extracellular domain (ECD) formed by two extracellular helices and two extracellular loops. The ECD faces the ER lumen (Fig. 1a; Supplementary Fig. S1c). Its dimensions are 63 Å × 33 Å × 34 Å, and the N- and C-termini are located on the lumen side and cytosol side, respectively (Fig. 1a, b). Overall, the charge distribution follows the positive-inside rule (Fig. 1c)^36^. The TMD consists of TM1–9, among which TM1–3 and TM7 form the core region, surrounded by the remaining TMs (Fig. 1b). TM1 is located in the center of the core region, consisting of two distinct segments: TM1a and TM1b. The N-terminus of TM1b extends 21 Å deep into the membrane plane, forming the bottom of the substrate-binding cavity alongside TM2 (Fig. 1b). The ECD contains two luminal loops (EL1 and EL2) that link TM2 to TM3 and TM6 to TM7, respectively. EL2 also includes an extracellular helix, namely EH2 (Fig. 1d–f). A conserved disulfide bond between C109 on EL1 and C245 on EL2 was identified, which likely contributes to maintaining the stabilization of ECD (Fig. 1d–f). The TMD and ECD together form an open positively charged cavity toward the ER side (Fig. 1c). Previous studies indicate that the members of the PAP2 family share a signature sequence ‘X6RPX–PSGHX–SRX5HX3Q’^37–39^, and this sequence is represented by three motifs within G6PC1: C1, ‘^76^KWILFGQRPY^85^’; C2, ‘^116^PSGHA^120^’; and C3, ‘^169^SRIYLAAHFPHQ^180^’ (Supplementary Figs. S6, S7a). The putative active center is formed by the signature motifs and locates within the cavity. *N*-linked glycosylation on N96 of G6PC1 is crucial for maintaining hydrolysis activity and is essential for the proper folding and stability of G6PC1 within the ER^40,41^. However, *N*-linked glycosylation is not observed in the G6PC1 structures, likely due to its high flexibility.

### Binding of G6P and conformational changes

**Fig. 2:**
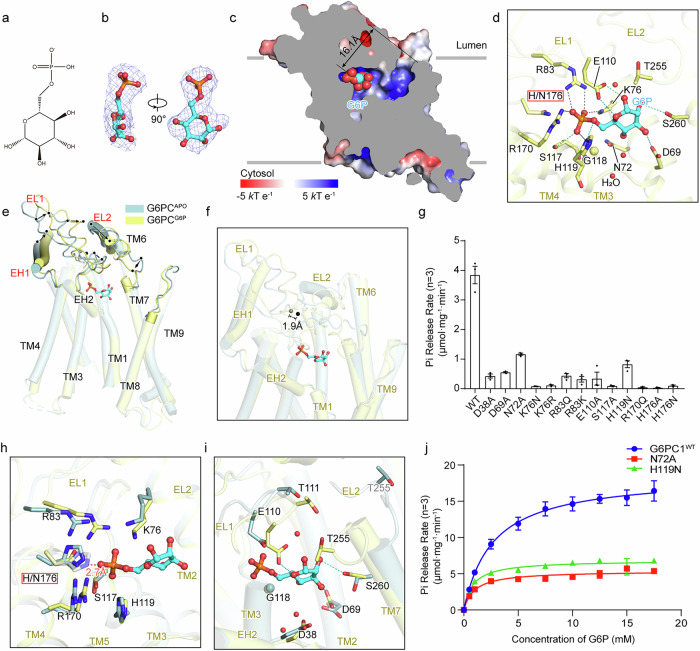
Substrate recognition and induced-fit conformational change of hG6PC1. **a** Formula of G6P. **b** Cryo-EM density and structure of G6P from different views. **c** Surface electrostatic potential of the G6PC1^G6P^ from a slice view through the binding pocket of G6P. G6P is demonstrated as spheres, the distance between G6P and the orifice of cavity is labeled. **d** Interactions between G6P and residues from G6PC1^G6P^. The hydrogen bonds and electrostatic interactions are depicted as dashed lines and colored teal and black, respectively. Interacting residues and G6P are shown as sticks and labeled. The carbon atoms of G6PC1 and G6P are colored pale yellow and cyan, respectively. Water molecules are represented as red spheres. **e** Structural comparison between G6PC1^G6P^ and G6PC1^APO^, which are colored as pale yellow and light blue, respectively. The significant conformational changes are labeled with red letters. G6P is shown as sticks. **f** Comparison of ECD COMs between G6PC1^APO^ (grey) and G6PC1^G6P^ (black). **g** Hydrolytic activity measured by the rate of phosphate release from WT G6PC1 and mutants solubilized in LMNG-cholesterol hemisuccinate (CHS). Background activity of post-denaturation was subtracted. Results are averages of three independent measurements, and error bars represent calculated standard error of the mean (*n* = 3). Pi phosphate. **h** Superposition of the phosphate-binding pocket between G6PC1^G6P^ and G6PC1^APO^. The distance between putative H176 and the phosphorus atom of G6P is demonstrated as a red dashed line and labeled. **i** Comparison of the glucose moiety-binding pocket between G6PC1^G6P^ and G6PC1^APO^. **j** Michaelis–Menten kinetics of G6P hydrolysis by G6PC1 and its variants. Michaelis–Menten kinetics of G6P hydrolysis indicates that the specific activity of variant N72A (V_max_ = 5.384 μmol/mg/min; *K*_m_ = 0.979 mM) and H119N (V_max_ = 6.843 μmol/mg/min; *K*_m_ = 0.838 mM) is lower than the WT G6PC1 (V_max_ = 18.610 μmol/mg/min; *K*_m_ = 2.673 mM) (*n* = 3).

G6P is a negatively charged molecule in cells and is involved in glucose metabolism pathways such as the pentose phosphate pathway and glycolysis^42^. It is formed by the phosphorylation of the C6 primary hydroxyl group of glucose and serves as the physiological substrate for G6PC1^43^ (Fig. 2a). It is widely accepted that the process of G6PC1 hydrolyzing G6P is initiated by a nucleophilic attack by histidine at site 176, resulting in the formation of a phosphohistidine bond and the breakdown of a carbonyl^44^. Therefore, we introduced the H176N mutation to block the initial step of hydrolysis to capture the state of G6PC1 bound to the substrate G6P before the beginning of the reaction. We successfully determined the complex structure of G6P and G6PC1^H176N^ at a 2.9 Å resolution, and cryo-EM maps revealed a local density that clearly defined G6P (Fig. 2b). We name this complex G6PC1^G6P^.

G6PC1^G6P^ forms a narrow, deep, positively charged cavity facing the ER lumen, accommodating G6P at its deepest position, 16.1 Å from the orifice (Fig. 2c). G6P is roughly oriented parallel to the membrane, with its phosphate group positioned at the bottom of the cavity and the glucose moiety near the opening, tilted toward the cytosol. Three cryo-EM densities (W1, W2, W3) in the active center are identified as water molecules (Supplementary Fig. S7b), which form a hydrogen bond network with nearby residues to stabilize G6P (Supplementary Fig. S7c, d). G6P interacts extensively with conserved residues from EL1, EL2, TM1, TM2, TM3, and TM4 (Fig. 2d; Supplementary Fig. S6). Charged residues, including R83^EL1^, K76^TM2^, H119^TM3^, R170^TM4^, form multiple salt bridges with G6P. The side-chain oxygen atoms of S117^EL1^, H119^TM3^, E110^EL1^, S260^TM7^, and D69^TM1^ and several main-chain nitrogen atoms stabilize G6P via hydrogen bonds (Fig. 2d; Supplementary Fig. S7f). The hydrogen bond between H119 and the bridging oxygen atom probably contributes to the protonation of the bridging oxygen atom during hydrolysis. In addition, G114 and G118, two conserved small side-chain residues in the PAP2 family, provide flexibility for the substrate-binding pocket (Supplementary Fig. S7g). These interactions position G6P favorably for nucleophilic attacks. Mutagenesis validated these findings: K76N, S117A, and R170Q abolish G6PC1 activity, while D38A, D69A, R83Q, and E110A retain ~10% activity (Fig. 2g). Both N72A and H119N significantly decrease enzymatic efficiency and impair the final catalytic effect (Fig. 2g, j). We propose that N72A and H119N probably constrain the G6PC1 and G6P complex in a reactive intermediate state, preventing the subsequent dephosphorylation. R83 and K76 orientations are crucially anchored by hydrogen bonds from G112 and P113 main-chain oxygen atoms (Supplementary Fig. S7e). Charge-preserving mutations (R83K, K76R) also eliminate hydrolysis, likely due to disrupted local spatial arrangements (Fig. 2g). Additionally, the profiles of fluorescence size exclusion chromatography (FSEC) on these mutations exhibit that K76R, D69A, R83K and R83Q mutations significantly impair the expression level of G6PC1, and D38A probably impairs both protein expression and normal folding (Supplementary Fig. S13a). To investigate the contribution of histidine at site 176, we modeled the amide group of N176 as an imidazole group, while preserving the primary orientation of main chain. The putative imidazole nitrogen (H176) is 2.7 Å from the phosphorus atom of the phosphate group, facilitating the formation of a phosphohistidine bond during hydrolysis (Fig. 2h). The H176A and H176N mutations completely disrupted G6PC1 activity (Fig. 2g), supporting the key role of H176 in G6P hydrolysis.

To explore the specific interaction between G6PC1 and F6P, we supplemented ~20 mM F6P during purification for cryo-EM determination. Purified G6PC1 presented the activity to hydrolyze F6P (Supplementary Fig. S1b). The cryo-EM map clearly shows the presence of F6P (Supplementary Fig. S8b), confirming that it occupies the same binding pocket as G6P does (Supplementary Fig. S8c, d). The conformation of G6PC1^F6P^ and interactions with F6P closely resemble those observed in G6PC1^G6P^ (Supplementary Fig. S8d–f). However, D38 and L73 stabilize F6P via hydrogen bonds and hydrophobic contacts, partially compensating for the smaller furan ring of F6P than the pyran ring of G6P (Supplementary Fig. S8a, f). Despite this, the change in the position of the epoxide oxygen atom disrupts the hydrogen bond with E110, diminishing the affinity between G6PC1 and fructose (Supplementary Fig. S8f). Additionally, the smaller furan ring of F6P increases its distance from the coordinating residues, further weakening the binding.

To illustrate the details of the substrate-induced conformational changes in G6PC1, we performed a structural alignment between G6PC1^APO^ and G6PC1^G6P^ using TMD as references. The TMD structures are nearly identical (root mean square deviation (RMSD) 0.504 Å over 1290 atoms), whereas the ECD undergo significant conformational changes and displays an ~1.9 Å displacement of the center of mass (COM) (Fig. 2e, f). According to the comparison, we infer that the binding of G6P attracts nearby residues, causing local conformational changes, which leads to shifts in EL1, EH1, and TM7 toward the active center through extensive interactions within the ECD. Notably, EL2 undergoes dramatic rotation toward the active center, which, together with other changes in ECD, reduces the volume of the substrate-binding pocket from 1651.0 Å^3^ to 1364.0 Å^3^ (Supplementary Fig. S9a–d). On the other hand, K76, R83, R170, and H176 significantly shift, resulting in strong electrostatic interactions that anchor the phosphate group (Fig. 2h). Although the overall topology is completely different (Supplementary Fig. S10a), the binding site of the phosphate group shows a highly conserved arrangement of residues across the PAP2 family (Supplementary Fig. S10b–g), implying that these members likely share a conserved catalytic mechanism. Both D69 and S260 show side-chain rotations toward the glucose moiety, whereas E110 and T255 undergo notable changes in spatial positioning, which together contribute to the stabilization of the glucose moiety (Fig. 2i). Additionally, the displacement of D38 and T111 results in two extra hydrogen bond networks to stabilize G6P. These residues form the binding pocket of glucose moiety together (Fig. 2i). These significant conformational changes fulfill the structural requirements for the reaction, such as substrate coordination and the exclusion of other substrates, and alter the local microenvironment of the active center. These findings indicate the vital role of substrate binding-induced conformational changes in the hydrolysis process.

### Mechanism of the catalysis and product release

**Fig. 3:**
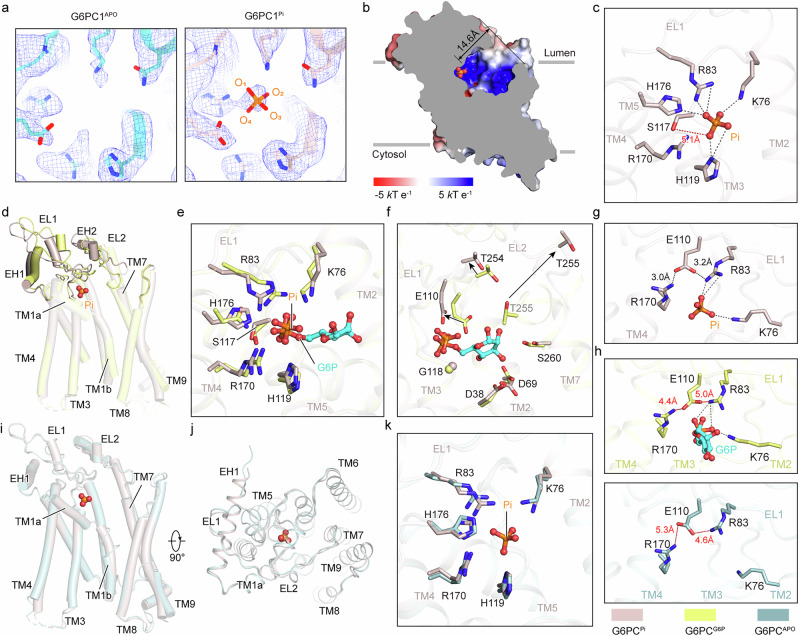
Mechanism of catalysis and product release. **a** Comparison of the cryo-EM density map of the phosphate-binding pocket between G6PC1^APO^ and G6PC1^Pi^. G6PC1^APO^ is colored light blue and shown on the left; G6PC1^Pi^ is colored light pink and shown on the right. Phosphate is depicted in orange. **b** Slice view of the electrostatic surface of G6PC1^Pi^. Phosphate is represented as spheres. The distance of phosphate from the cavity orifice is labeled. **c** Detailed interactions between G6PC1^Pi^ and phosphate. The interacting residues are shown as sticks. Phosphate is shown in a ball-and-stick representation. Phosphate and interacting residues are shown as opaque; the G6PC1 backbone are rendered translucent. Salt bridges are shown as black dashed lines. The non-existed interaction between S117 and the phosphate is depicted as a red dashed line and labeled. **d** Overall structural comparison between G6PC1^G6P^ and G6PC1^Pi^, which are colored pale yellow and light pink, respectively. Phosphate is represented as spheres. **e** Alignment of interactions in the phosphate-binding site between G6PC1^G6P^ and G6PC1^Pi^. **f** Comparison of interactions in the glucose moiety-binding site between G6PC1^G6P^ and G6PC1^Pi^. **g** Salt bridges between G6P and E110, R170 and R83 of G6PC1^Pi^. Side chains are shown as sticks, and electrostatic interactions are represented by black dashed lines. **h** Salt bridges between G6P and E110, R170 and R83 of G6PC1^APO^ and G6PC1^G6P^. The non-existed salt bridges are shown as red dashed lines and labeled. **i**, **j** Structural alignment between G6PC1^APO^ (light blue) and G6PC1^Pi^ (light pink), shown in side view (**i**) and top-down view (**j**). Phosphate is represented as spheres. **k** Comparison of interactions in phosphate-binding site between G6PC1^APO^ and G6PC1^Pi^.

To elucidate the specific mechanism of G6PC1 catalysis, we determined the complex structure of an inorganic phosphate bound to G6PC1 at 3.1 Å resolution (Supplementary Fig. S2), designated as G6PC1^Pi^. We observed an additional distinct cryo-EM density in the active center of G6PC1^Pi^ compared to G6PC1^APO^ (Fig. 3a). The cryo-EM map allowed us to place the phosphate within the density range, providing a clear visualization of the interactions between G6PC1 and the product (Fig. 3b, c). Phosphate adopts a highly symmetrical regular tetrahedron in real space because of the equal electronegativity of the four oxygen atoms, even though the four phosphorus‒oxygen bonds are not identical in chemical formula. Consequently, we numbered the oxygen atoms of phosphate as O_1_–O_4_ for clarity of description (Fig. 3a).

Following the hydrolysis reactions, the phosphate slightly shifted toward the lumen membrane and was in a strongly positively charged region 14.6 Å from the cavity orifice (Fig. 3b). The produced free phosphate is closer to R83 and K76 and is further stabilized by extensive interactions from TM2, TM3, TM4, TM5, and EL1 (Fig. 3c). Among them, R83, K76, H119, R170 and H176 exhibit a slight positional adjustment and form multiple salt bridges to stabilize the phosphate (Fig. 3c). The hydrolysis of G6P probably leads the ECD to undergo significant conformational changes to an open state, which facilitates the release of the products (Fig. 3d). Specifically, the hydrogen bond between S117 and the phosphate is broken, whereas R83, R170, and H176 shift slightly away from the phosphate, weakening phosphate binding. A previous study has indicated that the deprotonation of serine relies on the positively charged histidine in the Ser-His-Asp catalytic triad of the serine proteases^45^. Thus, we proposed a hypothesis: S117 likely acts as a proton donor to H176 during hydrolysis (Fig. 3c, e). The free phosphate is positioned slightly further from H176 than the phosphate group in G6P, suggesting that the glucose moiety in G6P likely contributes to the position of the phosphate group near H176 to facilitate hydrolysis (Fig. 3e). We observed that E110 forms two salt bridges with R83 and R170 — two residues which stabilize the phosphate (Fig. 3g). However, the two interactions are absent in the structures of both G6PC1^G6P^ and G6PC1^APO^ (Fig. 3h). This suggests that E110 may neutralize the electropositive charges of R83 and R170, thereby reducing their ability to stabilize phosphate binding. Additionally, the displacement of E110 away from the glucose moiety likely impairs their interaction (Fig. 3f). E110A completely eliminates the activity of G6PC1, underscoring its essential role in the hydrolysis process (Fig. 2g). Based on these observations, we propose that E110 plays a potential role in facilitating product release, which is consistent with the findings reported in previous studies^46,47^.

The positively charged environment of the phosphate-binding pocket intensely anchors the phosphate, confining the ECD in a membrane-proximal conformation through extensive interactions with the glucose moiety. Since G6PC1^APO^ represents the ground state, the open state of the ECD in this structure signifies its lowest energy state. During hydrolysis, the cleavage of the phosphoester bond separates G6P into glucose and phosphate, removing the constraint on the ECD. Consequently, the unrestricted ECD tends to return to its lowest energy state, adopting a conformation positioned further from the membrane (Fig. 3i–k). The movement of the ECD leads to significant displacement and rotation of the side chains within the glucose-binding pocket. Specifically, E110, T254 and T255 move away from the glucose, and D69 and S260 undergo rotations and slight displacements (Fig. 3f). These changes eliminate the extensive hydrogen-bonding interactions with the glucose and break the hydrogen bond networks centered around water molecules, facilitating glucose release. The freed glucose is then transported out of the cell by the glucose transporter^48^, ultimately contributing to the regulation of glucose homeostasis.

### Phosphatidylserine (PS) participates in the conformational changes of G6PC1

**Fig. 4:**
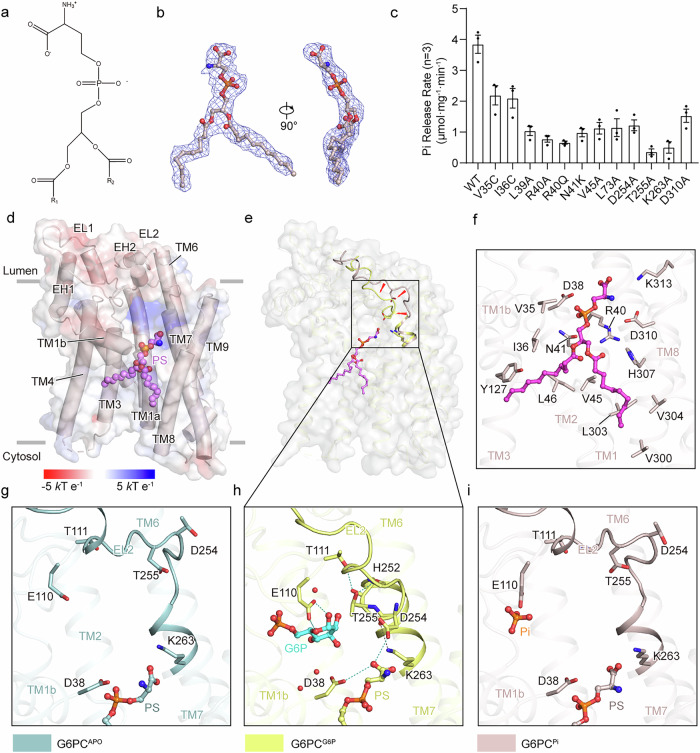
Mechanism by which PS regulates conformational changes. **a** Molecular formula of PS. **b** Structure and cryo-EM density of PS. **c** Hydrolytic activity measured by the rate of phosphate (Pi) release from WT G6PC1 and mutants solubilized in LMNG-CHS. Background activity of post-denaturation was subtracted. Results are averages of three independent measurements (*n* = 3), and error bars represent calculated standard error of the mean. **d** Overall structure of G6PC1^Pi^ in complex with PS. The surface electrostatic potential is shown as a translucent overlay. G6PC1^Pi^ is demonstrated as light pink cylinders. The structure of PS is depicted as spheres and colored by different atoms. **e** Conformational change of EL2 from G6PC1^G6P^ to G6PC1^Pi^. The overall structure is represented as translucent grey surface, and PS is depicted as sticks. G6PC1^G6P^ and G6PC1^Pi^ are colored pale yellow and light pink, respectively. **f** Detailed interactions between PS and G6PC1^Pi^. The interacting residues are shown as sticks, and PS are demonstrated in a ball-and-stick representation. The carbon atoms of PS are colored purple. **g**–**i** Comparison of interactions between the serine headgroup of PS and nearby residues in G6PC1^APO^ (**g**), G6PC1^G6P^ (**h**) and G6PC1^Pi^ (**i**). The hydrogen bonds are shown as teal dashed lines.

Lipids regulate the activity and stability of membrane proteins by influencing their conformation, function, and interactions. In all of the cryo-EM maps, a distinct and lipid-like density cradled by TM1, TM3 and TM8 always existed. To investigate the potential type of the lipid, we initially fitted four phospholipids into the density map and assessed their fit quality. Given that the primary discrepancy among them arises from the hydrophilic head group, we focused on evaluating the fit between the head groups and the density map. The head-group profile in the cryo-EM map appears flat, which favors the accommodation of the serine group of PS (Supplementary Fig. S11a). In contrast, the choline group of phosphatidylcholine (PC) adopts a regular tetrahedral shape, which would typically correspond to a subcircular profile in the cryo-EM map. While PS demonstrates a strong match with the cryo-EM map, PC only fits approximately (Supplementary Fig. S11b). The amine group of phosphatidylethanolamine (PE) is too small to align with the cryo-EM map, whereas the inositol group of phosphatidylinositol (PI) is too large to be accommodated by the density map (Supplementary Fig. S11c, d). The identification of phospholipid types was further supported by analyzing the interactions between the head groups of phospholipids and G6PC1. Several hydrophilic and charged residues within the binding pocket facilitate the formation of hydrogen bonds and salt bridges. The serine group of PS contains two oxygen atoms and one nitrogen atom, enabling it to form stable interactions with nearby residues (Supplementary Fig. S11e). In contrast, while the choline group of PC also features a hydrophilic nitrogen atom, most distances between it and the surrounding residues exceed 4.0 Å, which is unfavorable for forming stable interactions. Furthermore, the presence of four peripheral methyl groups prevents the nitrogen atom from forming strong interactions with G6PC1 (Supplementary Fig. S11f). Although the methyl groups are absent in PE, the distance between its nitrogen atom and the nearby residues is too large to stabilize binding (Supplementary Fig. S11g). While PI is capable of forming hydrogen bonds with surrounding residues, the bulky inositol group makes it unfavorable to model PI within this density map (Supplementary Fig. S11d, h). These observations support modeling PS within this density. To further validate the accuracy of PS modeling, we conducted molecular dynamics (MD) simulations to compare the stability of PS and PC binding to G6PC1. Our results showed that PS consistently binds to the same pocket of G6PC1 across all 8 simulation trajectories, with a similar binding pattern to that observed for G6P (Supplementary Fig. S12a). Furthermore, RMSD analysis of PS exhibited a significant binding stability compared to PC, supporting the PS modeling (Supplementary Fig. S12b–e). According to these results, we propose that the lipid density represents a PS molecule (Fig. 4b).

PS is a negatively charged phospholipid synthesized at the ER and enriched in the plasma membrane (PM) and late secretory/endocytic compartments^49^. PS consists of two fatty acids ester-linked to the first and second carbons of glycerol, with serine attached via a phosphodiester linkage to the third carbon (Fig. 4a). The amide headgroup of PS is stabilized by a hydrogen bond from D254 and a salt bridge from D38, whereas the phosphate group forms a strong electrostatic interaction with the guanidinium group of R40. A hydrophobic pocket formed by V35, I36, Y127, L46, and V45 stabilizes one of the fatty acid chains (Fig. 4f). Key interacting residues, particularly R40, form salt bridges with the phosphate group of PS, significantly contributing to the binding free energy, underscoring the accuracy of the PS-binding site (Supplementary Fig. S12f). Most of the residues bound with the head group of PS are similar in G6PC2 and G6PC3, while the residues interacting with the fatty acid chain are mostly non-conserved (Supplementary Fig. S6), suggesting a difference in PS binding affinity among subtypes.

To investigate the role of PS in G6P hydrolysis, we compared the structures of G6PC1^Pi^ and G6PC1^G6P^ based on the TMD, resulting in an RMSD of 0.507 Å over 1293 atoms. The comparison suggests that PS is likely directly engaged in stabilizing the partially open state of G6PC1^G6P^ (Fig. 4e). Specifically, the inward flipping of EL2 contributes to the generation of interactions between PS and G6PC1 (Fig. 4h). T255 facilitates EL2 to form a protruding fold through multiple hydrogen bonds with T111 and H252, which positions D254 closer to the PS (Fig. 4h). The carboxyl group of PS subsequently forms a hydrogen bond with D254, accompanied by an electrostatic interaction between K263 and the main-chain oxygen atom of D254, which together synergistically anchor EL2 at this position (Fig. 4h). Additionally, PS forms a hydrogen bond with D38 and participates in the W_2_–W_3_ hydrogen bond network, further facilitating the stable binding of G6P in the active center. Notably, these interactions are absent in the apo and phosphate-bound states, indicating that they are induced by G6P binding and play important roles in the subsequent hydrolysis process (Fig. 4g, i). To validate the functional effect of the observed PS interactions, we conducted a hydrolysis assay of G6PC1 mutations in the PS-binding pocket. Consistent with our observations, most of the mutations within the binding pocket severely disrupt the activity of G6PC1 (Fig. 4c). The FSEC profiles of these mutations indicate that all introduced mutations decrease the expression level of G6PC1, while both R40A and V35C impair the protein folding (Supplementary Fig. S13b). Therefore, given that mutations could alter the intrinsic property of the protein, we emphasize that the observed effects of mutations may result from a combination of disrupted lipid binding and alterations in catalytic efficiency or protein stability. Notably, replacements of residues directly engaged in conformational changes, such as D254A, T255A and K263A, almost completely abolish the enzymatic activity without causing significant negative effect on the protein expression or folding (Fig. 4c; Supplementary Fig. S13b), indicating that conformational changes have a decisive impact on hydrolysis.

### The specific mechanism underlying GSD-1a-causing mutations

**Fig. 5:**
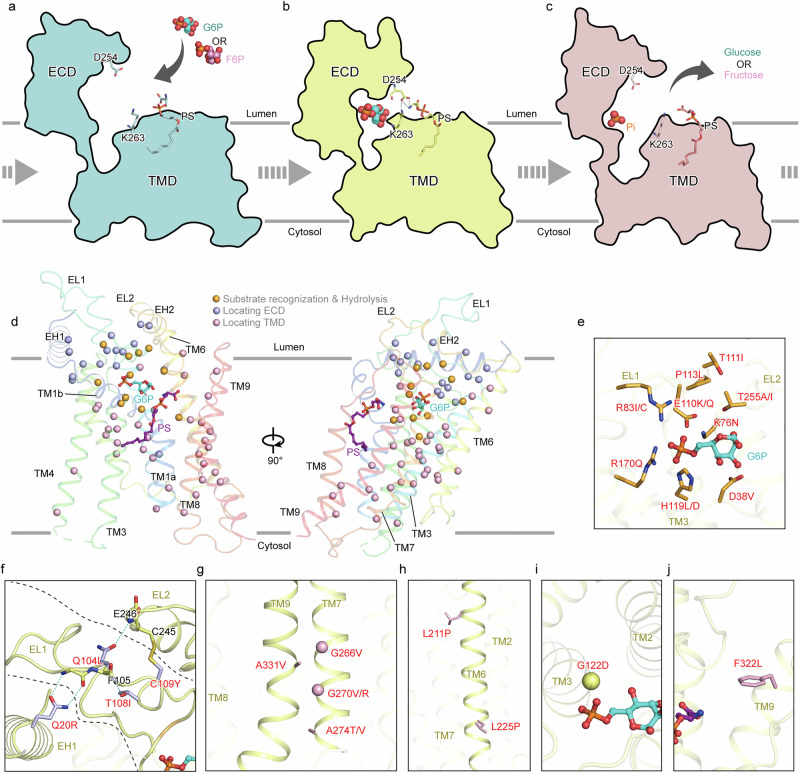
Conformational changes of G6PC1 and effects of disease-causing mutations. **a**–**c** Schematic diagram depicting the process of induced-fit conformational changes. The binding of substrate induces ECD to move near the membrane. PS, D254 and K263 stabilize the occluded conformation. The substrates and products are shown in a ball-and-stick representation. Different states are colored light blue, pale yellow and light pink for G6PC1^APO^, G6PC1^G6P^ and G6PC1^Pi^, respectively. **d** Distribution of disease-causing mutations in G6PC1. The structure of G6PC1 is shown as cartoon and colored by rainbow. Mutations are shown as spheres and colored by the different classes: orange for the mutations in residues contributing to G6P recognition and hydrolysis (class A); blue for the mutations in the ECD (class B) and pink for the mutations in the TMD (class C). The front view is shown on the left and the side view is exhibited on the right. **e** Detailed mutations in class A. The relative mutations are labeled with red letters. The residues are shown as sticks, and G6P is depicted in a ball-and-stick representation. **f** Examples for mutations in class B. The hydrogen bonds are demonstrated as teal dashed lines. The disease-causing mutations are labeled as red letters. **g**–**j** Several instances in class C of disease-causing mutations, shown as sticks and labeled with red letters.

Mutations in G6PC1 are the root cause of GSD-1a, and restoring G6PC1 activity is a fundamental approach for treating this condition. To date, more than 100 disease-causing mutations in G6PC1 have been identified in patients with GSD-1a, according to the Human Gene Mutation Database (Fig. 5d). Our work sheds light on the potential impact of these mutations on G6PC1 from a structural biology perspective. According to the structures, we roughly categorized these mutations into three classes. Class A represents mutations located in the G6P-binding site, probably disrupting substrate binding or hydrolysis (Fig. 5e). Mutations within the active center, such as K76N^50^, R83I/C^10,51^, H119L/D^52,53^, and R170Q^54^, abolish hydrolytic activity, underscoring their critical role in stabilizing G6P binding^10,55^. Notably, these mutations do not affect protein expression levels, indicating no impact on folding or overall stabilization^32,56^. Additionally, mutations like E110K/Q^57^ probably impair product release^46,47^. P113L significantly decreases G6PC1 expression level and causes gross misfolding^35^^,47^. Class B represents mutations located in the ECD (Fig. 5f), primarily affecting glycosylation, protein expression, and subcellular localization. For instance, Q20R^58^ and T108I^59^ disrupt glycosylation and abolish G6PC1 activity, whereas C109Y^60^ alters protein expression and subcellular localization^56,61^. Residues such as Q104^62^, T108, C109, and Q20 interact extensively, and mutations in these regions likely impair ECD stabilization (Fig. 5f). Other mutations, including M5R, T16A, T111I, W236R, A241T, T255I, and P257L, also reduce catalytic activity^12,56^. Class C represents mutations located in the TMD, likely disrupting proper folding or helix interactions. Most TMD mutations impair the stabilization of G6PC1^12,35^. For example, G118D, G125R, R149Q, and A331V do not affect protein expression levels but significantly reduce hydrolytic activity, likely due to their role in maintaining G6PC1 stabilization^13^. Structural analysis suggests that these mutations destabilize the tertiary structure of G6PC1 through steric hindrance (e.g., A331V^13^, G266V^63^, G270V/R^55,64^, and A274T/V^65,66^) or the introduction of proline residues that disrupt helices (e.g., L211P^57^ and L225P^67^) (Fig. 5g, h). Mutations such as G266V, G270V, and G270R have been shown to reduce protein synthesis levels, consistent with structural observations^56,68^. Additionally, a previous study proposed that TM3 is more flexible and tolerant of changes, based on the similar stabilization observed between the G122D/F322L mutations and the WT^12^. In our observations, G122 and F322 are located in the interhelical space, which likely better accommodate the moderate steric hindrance (Fig. 5i, j). Despite that, such mutations dramatically impair the enzymatic activity^69^, suggesting that they may affect the stability of G6PC1. These findings provide a reliable structural reference for the diagnosis of GSD1a, facilitating the development of targeted treatment strategies. The identified structural markers enhance diagnostic accuracy, enabling precise differentiation from other glycogen storage disorders. This advancement not only supports accurate diagnosis but also aids in the development of personalized therapies, potentially improving patient outcomes.

## Discussion

Human G6PC1 is a key enzyme in energy metabolism that is responsible for hydrolyzing G6P to free glucose and phosphate, thus regulating blood glucose homeostasis. Although extensive biochemical researches have provided insights into the physicochemical properties and biological functions of G6PC1, the comprehensive catalytic mechanism remains unclear. Additionally, the specific conformational changes induced by the interaction between G6PC1 and G6P are still uncertain. Here, we report the structures of G6PC1 in three distinct states during the catalytic process, providing comprehensive and detailed insights into the hydrolysis mechanism.

Specifically, G6PC1 resembles a ‘treasure chest’, with the ECD forming the lid and the TMD constituting the body; the active center is situated within this box (Fig. 5a–c). The binding of G6P induces a shift in the residues within the active center, further causing the ECD to move toward the membrane (Fig. 5b). This movement creates a narrow tunnel that prevents reactive G6P from leaking out and blocks other free G6P from entering, resulting in a relatively sealed microenvironment that favors the subsequent hydrolysis reaction. Additionally, the change in the ECD allows D254 to interact with PS and K263 in the TMD, effectively stabilizing the position of the ECD (Fig. 5b). Subsequently, G6P in the active center begins to undergo hydrolysis (Supplementary Fig. S14). The multiple positively charged residues near the phosphate group strongly attract electrons, reducing the electron density around the center phosphorus atom and making it positively charged, thereby favoring the proximity of the lone pairs of electrons on H176. The catalytic reaction is initiated by a nucleophilic attack of the basic nitrogen atom of the imidazole ring in H176 on the phosphorus atom of G6P, forming a five-covalent phosphorus intermediate. Immediately thereafter, the phosphoester bond between the phosphate group and the glucose breaks, decreasing the activation energy of the system. H119 provides a proton to the C6 negative oxygen to form a hydroxyl group. The cleavage of the phosphoester bond removes the constraints on the ECD. As the ECD returns to its ground state, it disrupts the glucose-binding pocket, resulting in the direct release of the glucose into the ER cavity. Moreover, an oxygen atom transfers its electron to form a double bond with the phosphate. The resulting phosphohistidine represents an intermediate state of the catalytic reaction. The histidine is subsequently replaced by a nucleophilic attack from a water molecule, leading to the formation of free phosphate. The negatively charged E110 likely engages in the release of products. The produced phosphate is released into the ER lumen, which facilitates the recovery of the active center residues. In fact, the produced phosphate occupies the active center of G6PC1, leading to a feedback inhibition of the enzymatic activity^70^. To promote efficient hydrolysis, these products must be released in a timely manner.

In summary, our study elucidates the molecular basis of substrate binding and conformational changes of G6PC1, as well as the direct effects of disease-causing mutations. Additionally, for the first time, we report the regulatory mechanism of PS on G6PC1. These findings provide a platform for rational drug design targeting GSD-1a and offer a drug design framework based on the PS-binding site, thereby opening broad prospects for effective treatment of glucose metabolism disorders.

## Materials and Methods

### G6Pase activity assay

For each G6PC1 construct, 50 mL of harvested cells were suspended in 10 mL of cold 20 mM Tris-HCl (pH 8.0), 150 mM NaCl, and 1 mM EDTA supplemented with 2 μM leupeptin, 0.8 μM pepstatin, and 2 μM aprotinin. Cell lysis was performed using a Dounce homogenizer. The membrane fraction was subsequently isolated by ultracentrifugation at 45,000× *g* for 40 min and immediately resuspended in 4 mL of solubilization buffer containing 20 mM Tris-MES (pH 6.5), 50 mM NaCl, 1 mM EDTA, 1% (w/v) LMNG-CHS, 2 μM leupeptin, 0.8 μM pepstatin, and 2 μM aprotinin, followed by agitation for 1 h. Insoluble debris was removed by a second round of ultracentrifugation at 45,000× *g* for 40 min. The resulting supernatant was aliquoted, quick-frozen in liquid nitrogen, and stored at –80 °C for further use.

G6Pase enzymatic activity was assessed by measuring the release of inorganic phosphate from G6P via Taussky and Shorr’s method (Taussky and Shorr, 1953). To initiate hydrolysis, 5 μL of the solubilized enzyme mixture was diluted with 50 μL of hydrolysis reaction buffer containing 1.5 mM G6P and incubated at 37 °C for 3 min, a duration optimized on the basis of preliminary experiments ensuring the linearity of hydrolysis reaction. The reaction was terminated by adding 50 μL of 12% (w/v) SDS. For the blank controls, 12% (w/v) SDS was added to the enzyme mixture before the hydrolysis reaction was initiated.

To quantify phosphate release, 60 μL of the terminated reaction mixture was transferred to a new transparent flat-bottom 96-well plate and mixed with 140 μL of preprepared Taussky and Shorr color reagent consisting of a 6:1 mixture of 0.42% ammonium molybdate and 10% (w/v) ascorbic acid in 0.5 M sulfuric acid. After incubation at 37 °C for 5 min, color development was assessed by measuring the absorbance at 820 nm. Phosphate concentrations were determined using a standard curve of phosphate standards.

To normalize for expression level variations of mutations, we employed FSEC and generated a standard curve reflecting the concentration of protein and FSEC peak height to calculate the protein quantity. The absolute amount of released phosphate was normalized to protein quantity and reaction time. The resulting catalytic activities are reported as ‘phosphate release rate’, with a unit of ‘μmol·mg^–1^·min^–1^’. For the Michaelis–Menten kinetics measurement of tagged and untagged G6PC1, the absolute protein quantity could not be measured because the untagged G6PC1 lacks fluorescence. Therefore, we represented the activity of them with ‘relative phosphate release rate’ (nmol·min^–1^). All reactions were performed in triplicate. Statistical error propagation was performed accordingly.

### Cloning, expression and purification of human G6PC1

The human full-length *G6PC1* gene (UniProt ID: P35575) was amplified from the HEK293F cDNA library using polymerase chain reaction (PCR) and subcloned and inserted into a PEG BacMam vector via a phata DNA polymerase kit (Vazyme, China). A GFP tag with a twin strep tag was fused at the C-terminus of full-length G6PC1, and an HRV-3C protease site was inserted between GFP and G6PC1. G6PC1 was expressed using baculovirus-mediated transduction of mammalian HEK293F cells^71^. When the number of HEK293F cells reached 2.5 × 10^6^ per mL, 2% P3 (the third generation) recombinant baculovirus (v/v) and 1% fetal bovine serum (v/v) were added to the cells, which were subsequently incubated at 37 °C with 5% CO_2_ for 12 h to induce expression. Subsequently, 10 mM sodium butyrate (Sigma, USA) was added to the cells, and the cells were cultured at 30 °C in 5% CO_2_. After shaking at 200 rpm for 48 h, the cells were collected via centrifugation at 3000 rpm for 3 min and stored at –80 °C.

For G6PC1 purification, the cells were first thawed on ice and resuspended in buffer A (20 mM Tris-HCl, 150 mM NaCl (pH 8.0), 5 mM β-ME, 2 μg/mL aprotinin, 1.4 μg/mL leupeptin, and 0.5 μg/mL pepstatin A). After homogenization, the membrane was harvested by ultracentrifugation at 100,000× *g* for 30 min at 4 °C, followed by resuspension and solubilization in buffer A supplemented with 2 mM ATP, 5 mM MgCl_2_, 1% (w/v) LMNG and 0.15% (w/v) CHS for 2 h at 4 °C. The mixture was centrifuged at 100,000× *g* for 30 min to remove insoluble debris. The supernatant was then suction-filtered and further purified via Strep-Tactin affinity resin (Smart-Lifesciences, China). The resin was washed with 5 column volumes (CVs) of buffer B (20 mM Tris-HCl, 150 mM NaCl, pH 8.0; 5 mM β-ME; 2 mM ATP; 5 mM MgCl_2_; 0.01% (w/v) LMNG; and 0.0015% (w/v) CHS) to remove the nonspecific binding proteins. GFP-tagged G6PC1 was eluted with 2 CVs of buffer B supplemented with 50 mM D-biotin. After that, the protein was concentrated and further purified via size-exclusion chromatography on a Superose 6 Increase 10/300 GL column (GE Healthcare, USA). The peak fractions were pooled and concentrated to at least 2 mg/mL for subsequent nanodisc reconstitution.

### Nanodisc reconstitution

Before reconstitution, soybean lipids were solubilized in TBS buffer (20 mM Tris-HCl, 150 mM NaCl, pH 8.0) supplemented with 2% (w/v) LMNG at 4 °C for 30 min. Freshly purified GFP-tagged G6PC1 protein, MSP1D1, and solubilized soybean lipids were then mixed at a ratio of 1:5:100 and incubated together at 4 °C for 1 h. The SM-2 bio-Beads (600 mg/mL) were subsequently added to the mixture and rotated overnight at 4 °C to remove the detergent. The next day, the mixture was centrifuged at 15,000 rpm at 4 °C to remove the Bio-Beads, and the supernatant was passed through a Streptactin Bead 4FF and washed with 10 CVs of TBS buffer to eliminate empty nanodiscs that do not contain the target proteins. For untagged sample preparation, reconstituted GFP-tagged G6PC1 was then eluted with 2 CVs of TBS buffer containing 50 mM biotin, and the GFP and twin strep tags were cleaved via prescission protease for 1 h at 4 °C. The prescission protease was subsequently removed via a Ni-NTA column. The flow-through was collected and concentrated to a volume of less than 1 mL, followed by size-exclusion chromatography on a Superose 6 Increase 10/300 GL column to remove GFP and the twin strep tags. The peak fractions were pooled and concentrated to approximately 10 mg/mL. To prepare the G6PC1^G6P^, G6PC1^F6P^, and G6PC1^Pi^ samples, 10 mM G6P, 20 mM F6P, or 10 mM Na_3_PO_4_ were each incubated with the protein for 30 min at 4 °C before cryo-EM sample preparation.

### Cryo-EM sample preparation and data collection

A droplet of 2.5 μL of 10 mg/mL freshly purified G6PC1 complex was applied to holey carbon grids (Cu R1.2/1.3 300 mesh; Quantifoil, USA) grow-discharged under H_2_–O_2_ conditions for 60 s using a Solarus plasma cleaner (Gatan, USA). The grid was then blotted with a blot force of 2, 4.5 s of blotting time and a 10.0 s wait time at 4 °C under an atmosphere of 100% humidity using Vitrobot Mark IV (Thermo Fisher Scientific, USA), followed by flash freezing in liquid ethane cooled with liquid nitrogen. Double blotting was used to increase the particle concentration in the micrographs. Cryo-EM data were acquired on a 300-kV Titan Krios G4 (Thermo Fisher Scientific, USA) with a Quantum GIF energy filter at a slit width of 10 eV, which was equipped with a K3 direct electron detector (Gantan, USA). Cryo-EM data were automatically recorded at 105,000× magnification in superresolution mode with a physical pixel size of 0.85 Å/pix via the EPU software package (Thermo Fisher Scientific, USA). Each movie was dose-fractionated to 32 frames at a dose rate of 15 e^–^/pix/s, resulting in a total dose of 60 e^–^/Å^2^. Defocus values range from –1.0 μm to –2.0 μm.

### Cryo-EM data processing

For the G6PC1^Pi^ dataset, 2213 movie stacks were collected. The data were processed in CryoSPARC. Patch Motion Correction was used for beam-induced motion correction, and patch contrast transfer function (CTF) estimation was applied to estimate the CTF. A total of 1,405,043 particles were initially picked via the blob picker and extracted with an unbinned box size of 256 pixels. Multiple rounds of 2D classification were performed to eliminate poor-quality particles sufficiently. The remaining 126,969 particles were reconstructed via ab-initio reconstruction in 3 classes to generate an initial map for subsequent heterogeneous refinement. The original 1,405,043 particles were then classified via multireference heterogeneous refinement, with the G6PC1 initial map as a good class and three distinctly shaped maps as junk classes. The good class subsequently underwent iterative ab-initio reconstruction, yielding 151,377 good particles. These particles were further refined and classified in 2D, with some distinct classes selected for Topaz Train. After that, 868,531 particles were re-extracted with an unbinned box size of 320 pixels and further classified via seed-facilitated multireference heterogeneous refinement. The good class was subjected to 2D classification to generate 348,168 good particles, and an additional 2 rounds without alignment 3D classification were conducted to yield 49,677 good particles. These particles underwent NU refinement and ultimately produced a 3.1 Å map on the basis of the gold-standard Fourier shell correlation criterion. G6PC1^APO^, G6PC1^G6P^ and G6PC1^F6P^ were performed via a similar procedure, and detailed flowcharts are shown in Supplementary Figs. S3, S4, and S5.

### Model building

The model of G6PC1^Pi^ was built de novo from a globally sharpened 3.1 Å map via the cryo-EM density map tool in COOT^72^. Using the large side chain as a benchmark, helices were manually fitted into the density map via COOT. After manual adjustment, the model was subjected to real space refinement against the final map generated by the secondary structure, geometry restraints and Ramachandran restraints in PHENIX^73^. Other models were generated via a similar procedure in COOT and PHENIX, relying on the model of G6PC1^pi^ as an initial model.

All figures were prepared with ChimeraX^74^ and PyMOL (Schrödinger).

### MD simulations

To confirm the binding of G6PC1 with PS and G6P, we conducted MD simulations on the G6PC1^G6P^ structure. We constructed the initial simulation system, which comprised a phospholipid bilayer membrane containing proteins, POPCs, and ligand molecules. In this setup, G6PC1 and lipids were parameterized using the Amber ff14SB force field^75^, water molecules were modeled with the TIP3P water model^76^, and ligands were assigned parameters from GAFF2^77^. The system was built using CHARMM-GUI^77^. The system included 150 mM NaCl to mimic physiological salt concentration and to maintain charge neutrality. Energy minimization was executed using the steepest descent approach. Pre-equilibration steps adhered to the standard CHARMM-GUI protocols. After equilibration, three productive independent simulations, each lasting 200 ns, were performed. Simulations were conducted under an NPT ensemble, maintaining a constant temperature of 303.15 K and a pressure of 1 bar. Temperature control was achieved through the v-rescale thermostat^78^, while the C-rescale barostat was used for pressure regulation^79^. The equations of motion were integrated using the Verlet leapfrog algorithm with a time step of 2 fs. Periodic boundary conditions were applied throughout. Lennard–Jones interactions and the real-space component of the Ewald summation were calculated with a cutoff distance of 0.9 nm, while the Fourier space component of the Ewald summation was handled using the particle mesh Ewald (PME) method^80^. All simulations were executed using Gromacs 2021.6^81^. Trajectory clustering analysis was performed using the GROMOS algorithm^82^. The calculation of RMSD for proteins and ligands was performed using VMD^83^. The MM/GBSA calculations were performed using the gmx_MMGBSA software, based on the final 100 ns of three independent trajectories^84^.

## Supplementary information

Supplementary Informations

## Data Availability

The three-dimensional cryo-EM density maps of human G6PC1^APO^, G6PC1^G6P^, G6PC1^Pi^ and G6PC1^F6P^ have been deposited into the Electron Microscopy Data Bank (EMDB) under accession numbers EMD-61811, EMD-61812, EMD-61813, and EMD-61814, respectively. The coordinates of human G6PC1^APO^, G6PC1^G6P^, G6PC1^Pi^, and G6PC1^F6P^ have been deposited into the Protein Data Bank (PDB) under accession codes 9JTL, 9JTM, 9JTN and 9JTO, respectively.
