# Supplementary material for: The induced-fit and catalytic mechanisms of human G6PC1
Source: Cell Discov. 2025 Jul 15;11:62. doi: 10.1038/s41421-025-00814-z (PMC12264158; doi:10.1038/s41421-025-00814-z)
Supplement: Supplementary file 1 — Supplementary Informations [file 41421_2025_814_MOESM1_ESM.pdf]

Supplementary Information for

The induced-fit and catalytic mechanisms of human G6PC1

This document contains Supplementary Fig. S1–S14 and Supplementary Table S1.

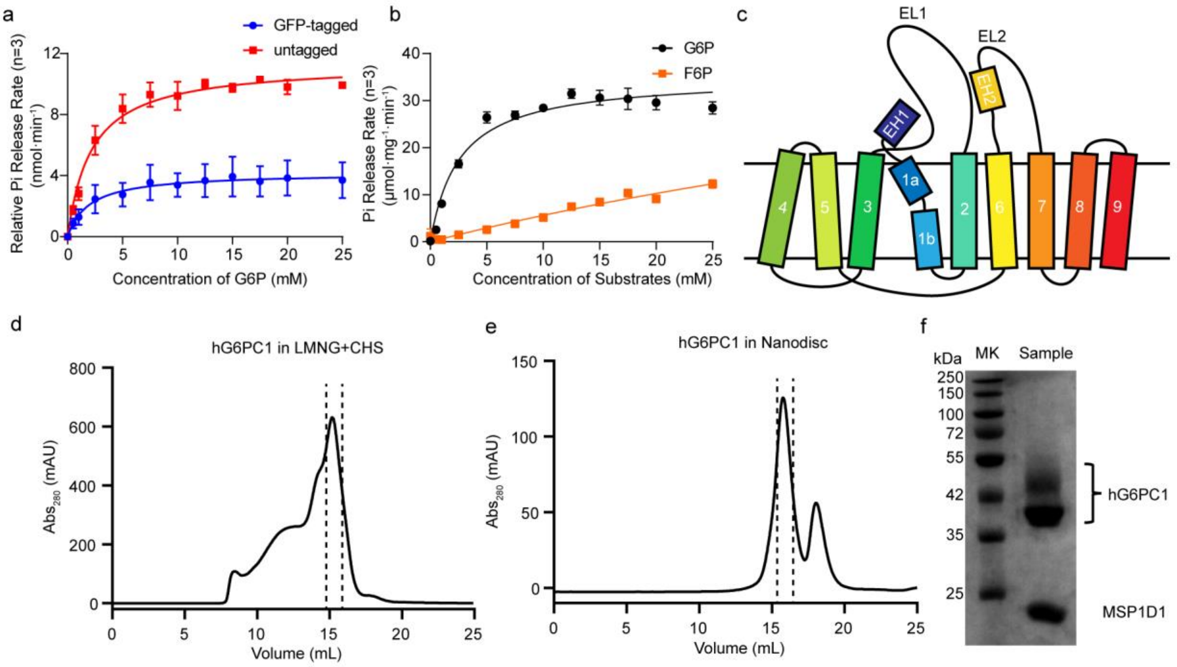

Supplementary Fig. S1 Functional representation and purification of *human* G6PC1.

a Kinetic analysis of GFP-tagged and untagged wild-type G6PC1. Solid lines are nonlinear least squares fits of the velocity data assuming a Michaelis-Menten model. The Km values of GFP-tagged and untagged G6PC1 are 1.985 mM and 2.186 mM, respectively; p value > 0.05 with unpaired t test, no statistically difference. **b** Kinetic analysis of G6PC1-GFP which is reconstituted into nanodiscs catalyzing G6P and F6P hydrolysis. Solid lines are nonlinear least squares fits of the velocity data assuming a Michaelis-Menten model. The K_m values of G6PC1 binding with G6P and F6P are 2.573 mM and 97.020 mM, respectively. The V_{max} values of them are 35.050 μmol/mg/min and 60.080 μmol/mg/min, respectively. The measurement was performed three times in different biology days. **c** Topology diagram of G6PC1. The helices and loops are labelled. The helices are colored in a rainbow spectrum. **d–e** The Representative profile of G6PC1 in LMNG+CHS (d) or nanodiscs (e) by size-exclusion chromatogram (Superose 6 Increase). Collected peak fractions for cryo-EM sample are highlighted with black dashed lines. **f** Representative SDS-PAGE result of cryo-EM sample with Coomassie blue staining. Marker is labelled on the left and proteins are labelled on the right. Glycosylation causes the extra electrophoretic band of G6PC1.

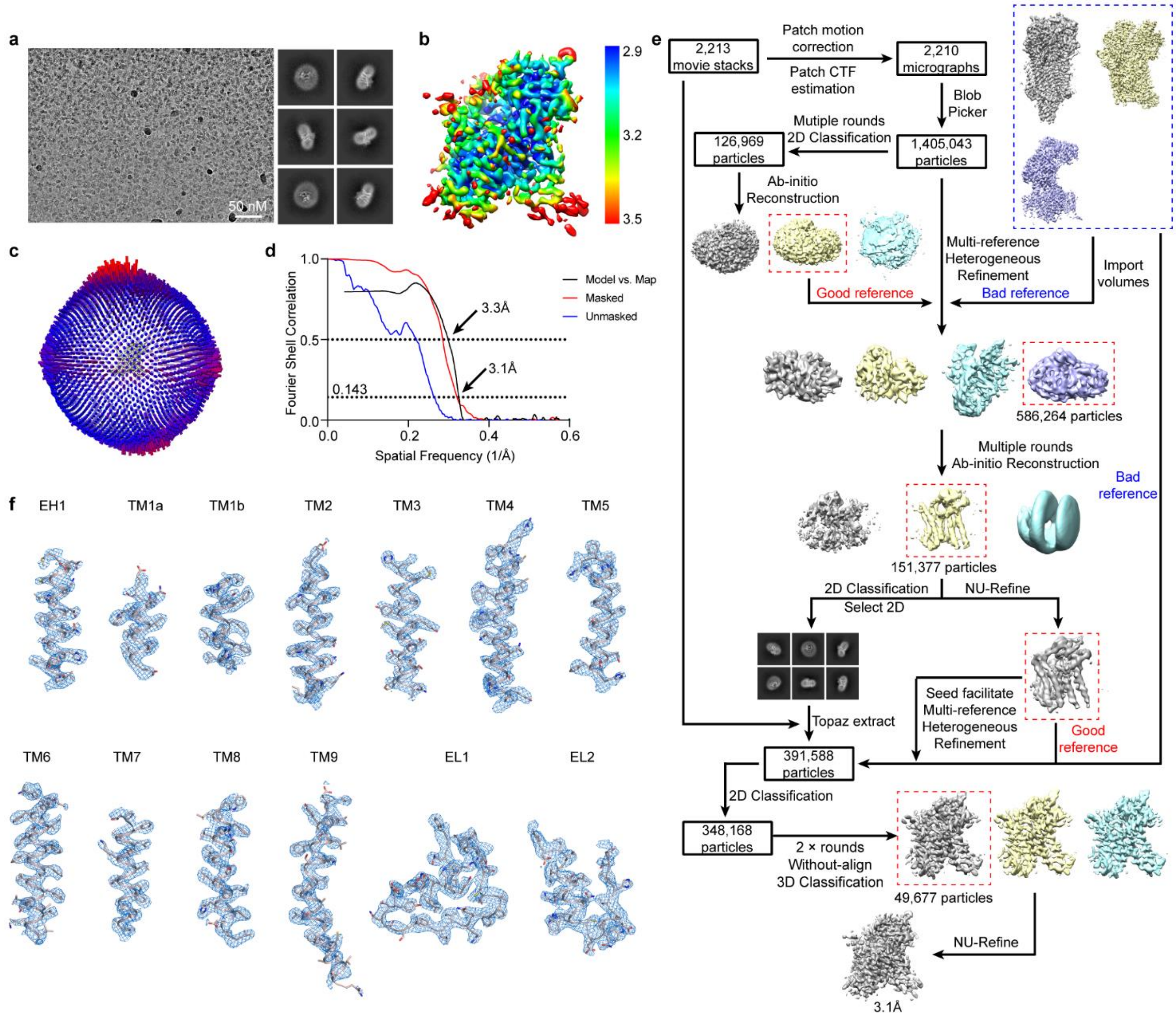

Supplementary Fig. S2 Cryo-EM data process of G6PC1^{Pi}.

a Representative motion-corrected micrograph (left) and 2D class results (right) of G6PC1^{Pi}. Scale bar of micrograph is 50 nm. **b** Local resolution distribution of G6PC1^{Pi}. The resolutions range from 2.9 Å to 3.5 Å, with the color gradient transitioning from blue to red. **c** Angular distribution of particles for the final reconstruction. The length of each spike represents the number of particles in the orientation. **d** Fourier shell correlation (FSC) curves of the final map. The curves of half maps before or after post-processing are colored blue and red, respectively. The curve of model-map is colored black. **e** Flow chart for cryo-EM data process of G6PC1^{Pi}. **f** Representative cryo-EM density maps for G6PC1^{Pi}.

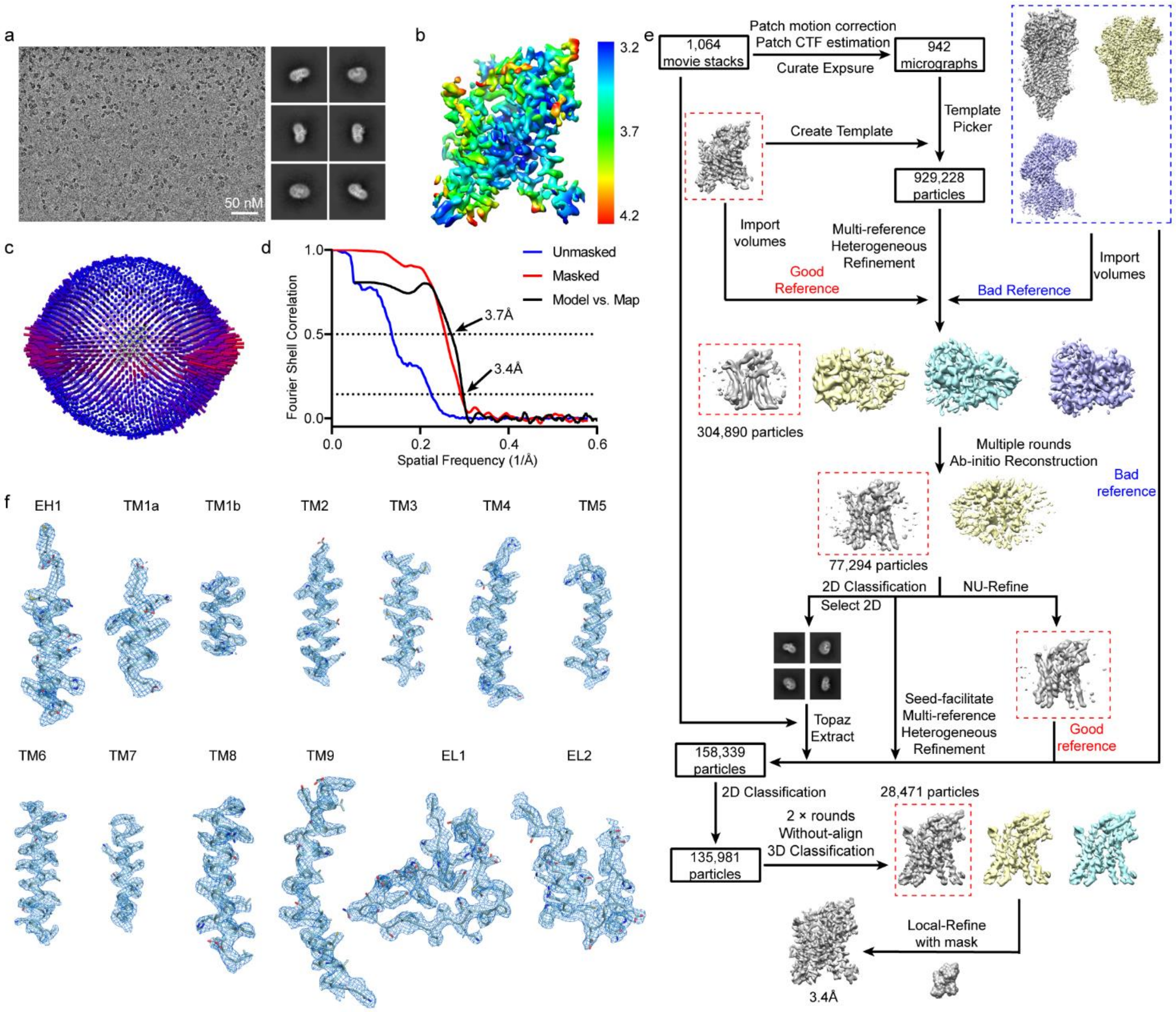

Supplementary Fig. S3 Cryo-EM data process of G6PC1^{APO}.

a Representative motion-corrected micrograph (left) and 2D class results (right) of G6PC1^{APO}. Scale bar of micrograph is 50 nm. **b** Local resolution distribution of G6PC1^{APO}. The resolutions range from 2.3 Å to 4.2 Å, with the color gradient transitioning from blue to red. **c** Angular distribution of particles for the final reconstruction. The length of each spike represents the number of particles in the orientation. **d** Fourier shell correlation (FSC) curves of the final map. The curves of half maps before or after post-processing are colored blue and red, respectively. The curve of model-map is colored black. **e** Flow chart for cryo-EM data process of G6PC1^{APO}. **f** Representative cryo-EM density maps for G6PC1^{APO}.

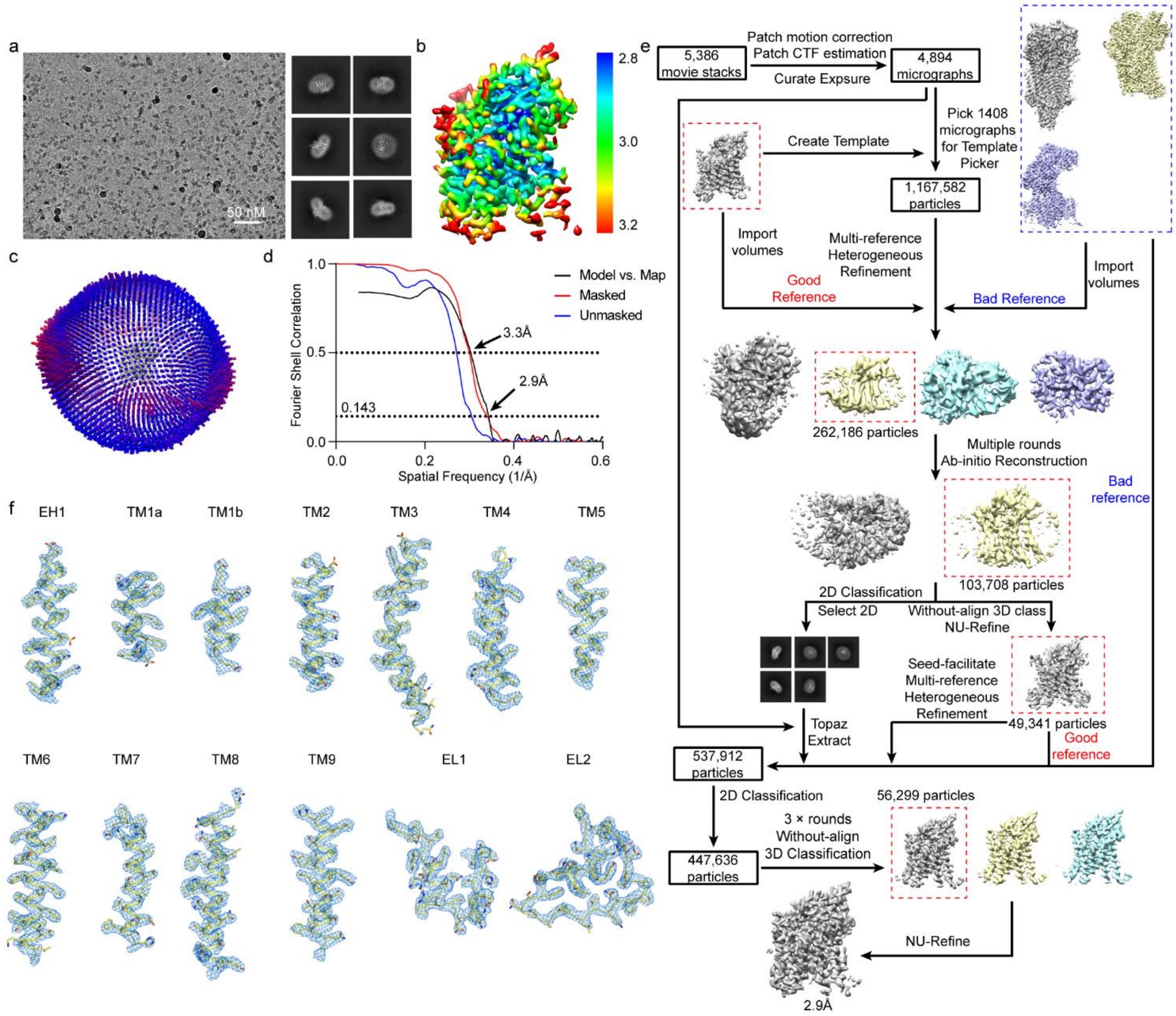

Supplementary Fig. S4 Cryo-EM data process of G6PC1^{G6P}.

a Representative motion-corrected micrograph (left) and 2D class results (right) of G6PC1^{G6P}. Scale bar of micrograph is 50 nm. **b** Local resolution distribution of G6PC1^{G6P}. The resolutions range from 2.8 Å to 3.2 Å, with the color gradient transitioning from blue to red. **c** Angular distribution of particles for the final reconstruction. The length of each spike represents the number of particles in the orientation. **d** Fourier shell correlation (FSC) curves of the final map. The curves of half maps before or after post-processing are colored blue and red, respectively. The curve of model-map is colored black. **e** Flow chart for cryo-EM data process of G6PC1^{G6P}. **f** Representative cryo-EM density maps for G6PC1^{G6P}.

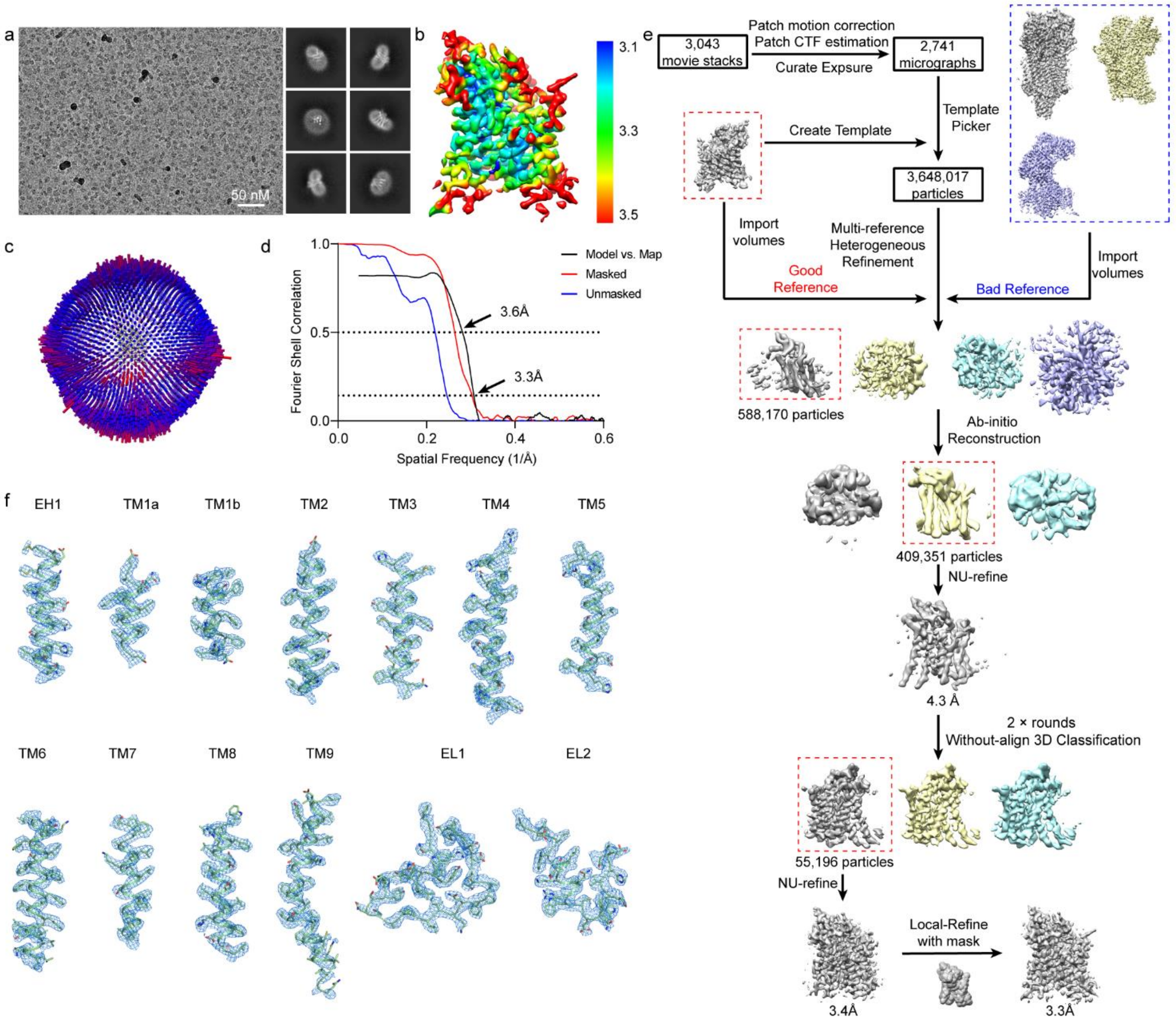

Supplementary Fig. S5 Cryo-EM data process of G6PC1^{F6P}.

a Representative motion-corrected micrograph (left) and 2D class results (right) of G6PC1^{F6P}. Scale bar of micrograph is 50 nm. **b** Local resolution distribution of G6PC1^{F6P}. The resolutions range from 3.1 Å to 3.5 Å, with the color gradient transitioning from blue to red. **c** Angular distribution of particles for the final reconstruction. The length of each spike represents the number of particles in the orientation. **d** Fourier shell correlation (FSC) curves of the final map. The curves of half maps before or after post-processing are colored blue and red, respectively. The curve of model-map is colored black. **e** Flow chart for cryo-EM data process of G6PC1^{F6P}. **f** Representative cryo-EM density maps for G6PC1^{F6P}.

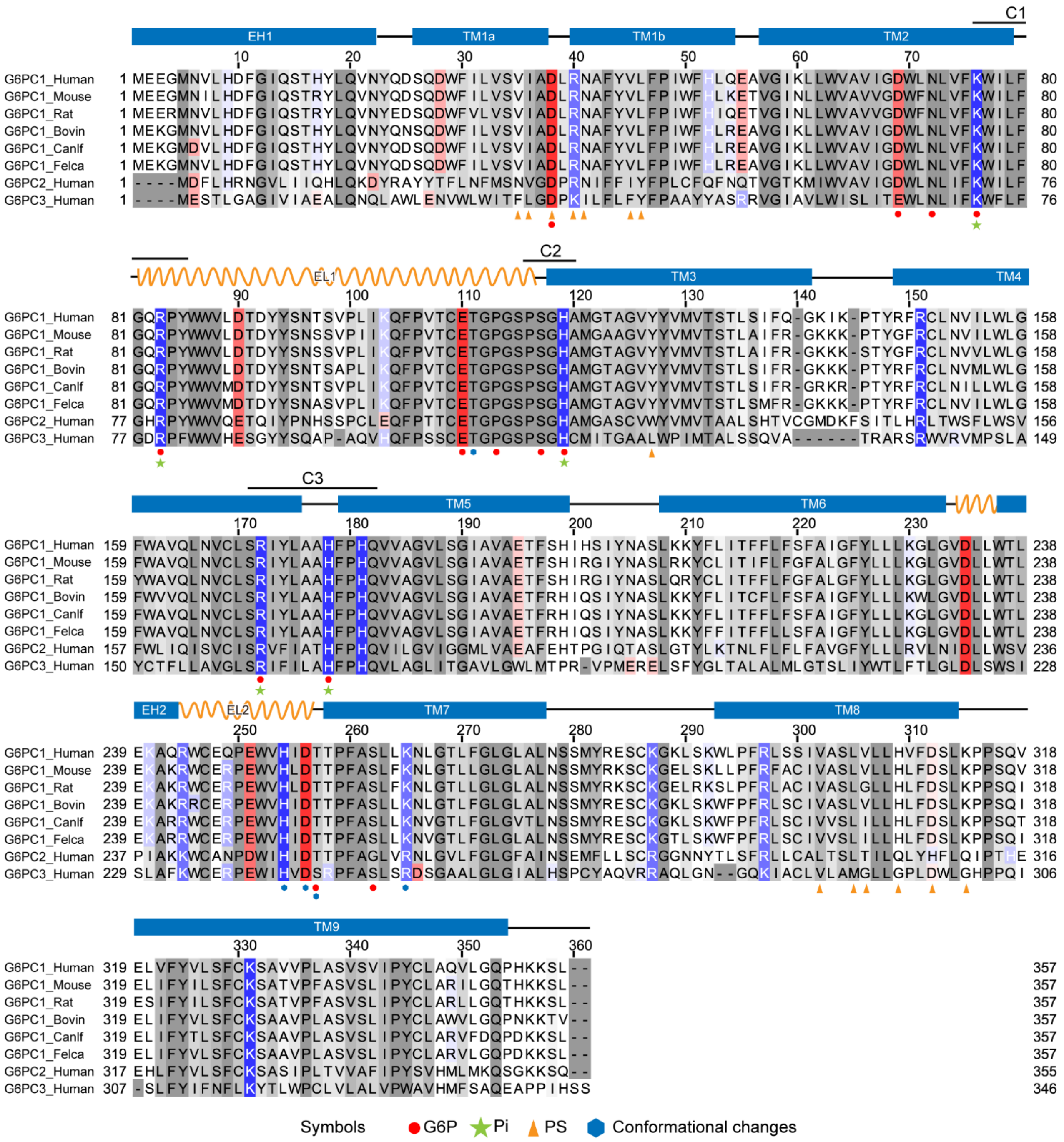

Supplementary Fig. S6 Sequence alignment of glucose-6-phosphatase catalytic subunits.

Sequence alignment of glucose-6-phosphatase catalytic subunits from variant species and subtypes. The conserved residues are highlighted, with negatively charged residues colored red and positively charged residues colored blue. Three conserved motifs (C1–C3) are labelled above the sequence. The helices are indicated as blue rectangles and the loops are depicted as orange wavy lines. The red circle, green star, orange triangle, and blue hexagon represent the conserved residues that interact with G6P, phosphate, PS, and are involved in conformational changes, respectively.

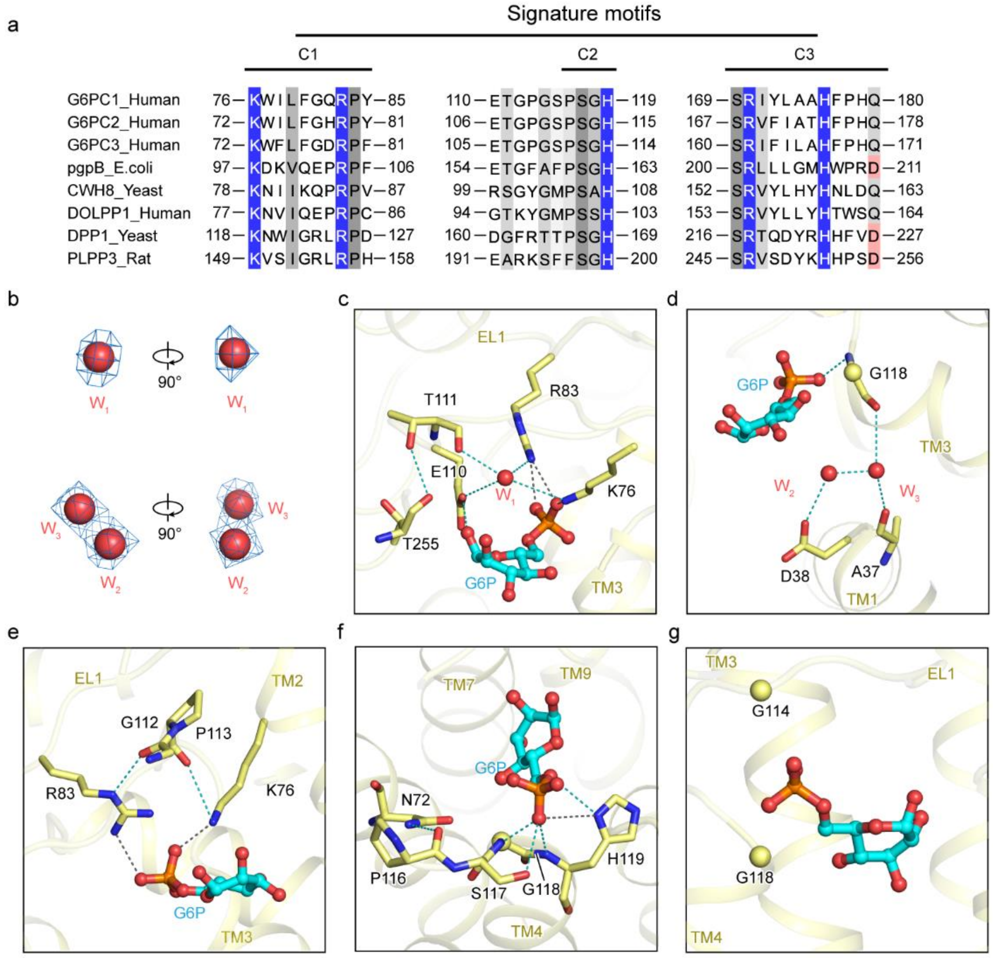

Supplementary Fig. S7 Signature motifs in PAP2 family and the detailed interactions between G6PC1^{G6P} and G6P.

a Sequence comparison of signature motifs in several members of PAP2 family. The conserved positively and negatively charged residues are colored blue and red, respectively. The C1–C3 motifs are labelled. **b** The Cryo-EM maps of water molecules. **c** Zoomed-in view of W_1 hydrogen bond network. The hydrogen bonds and electrostatic interactions are colored teal and black, respectively. Interactive residues and G6P are shown as sticks. The carbon atoms of G6PC1 and G6P are colored paleyellow and cyan, respectively. Water molecules are represented as red spheres. The cryo-EM density of W_1 water is depicted as blue mesh. **d** Zoomed-in view of W_2-W_3 hydrogen bond network. The cryo-EM densities of W_2 and W_3 water are depicted as blue mesh. **e** Interactions of R83 and K76 with nearby residues. **f** Interactions between phosphate group and the mainchain nitrogen atoms. **g** Relative positions of G118 and G114. The Ca atoms of them are shown as spheres.

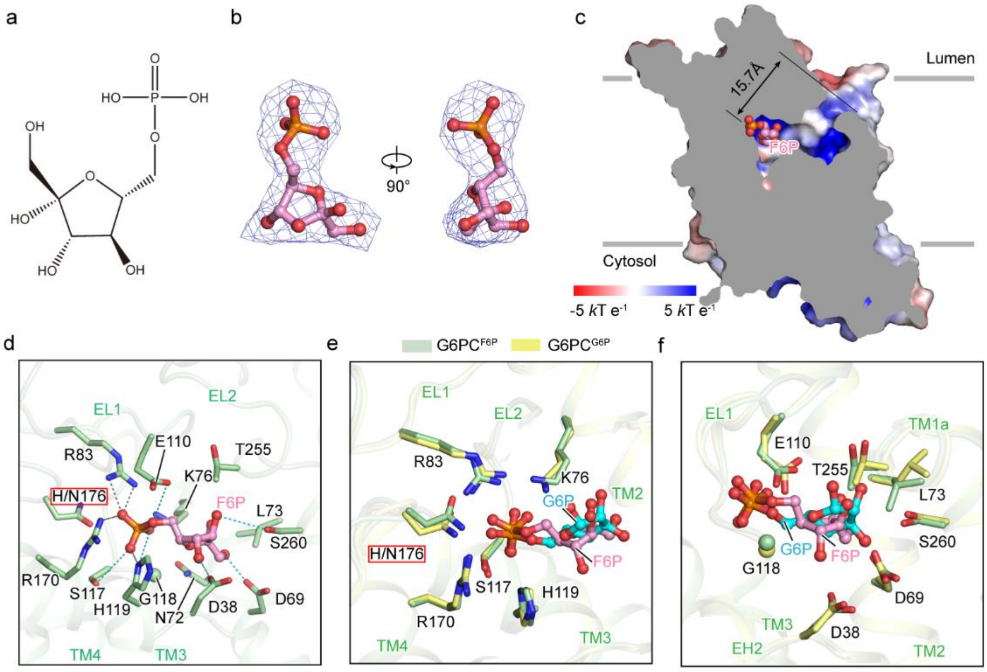

Supplementary Fig. S8 Fructose-6-phosphte binding site of hG6PC1

a Formula of fructose-6-phosphte (F6P). **b** Cryo-EM density map and structure of F6P, presented from different views. **c** Surface electrostatic potential of the G6PC1^{F6P} from in a slice view through the binding pocket of F6P. F6P is demonstrated as spheres, the distance between F6P and the orifice of cavity is labelled. **d** Interactions between hG6PC1 and F6P, characterized by dashed lines. The hydrogen bonds and electrostatic interactions are colored teal and black, respectively. Interactive residues and F6P are shown as sticks. The carbon atoms of G6PC1 and F6P are colored palegreen and pink, respectively. **e** Superpose of phosphate binding pocket between G6PC1^{F6P} and G6PC1^{G6P}, colored in palegreen and paleyellow, respectively. **f** Comparison of fructose moiety binding pocket between G6PC1^{F6P} and G6PC1^{G6P}.

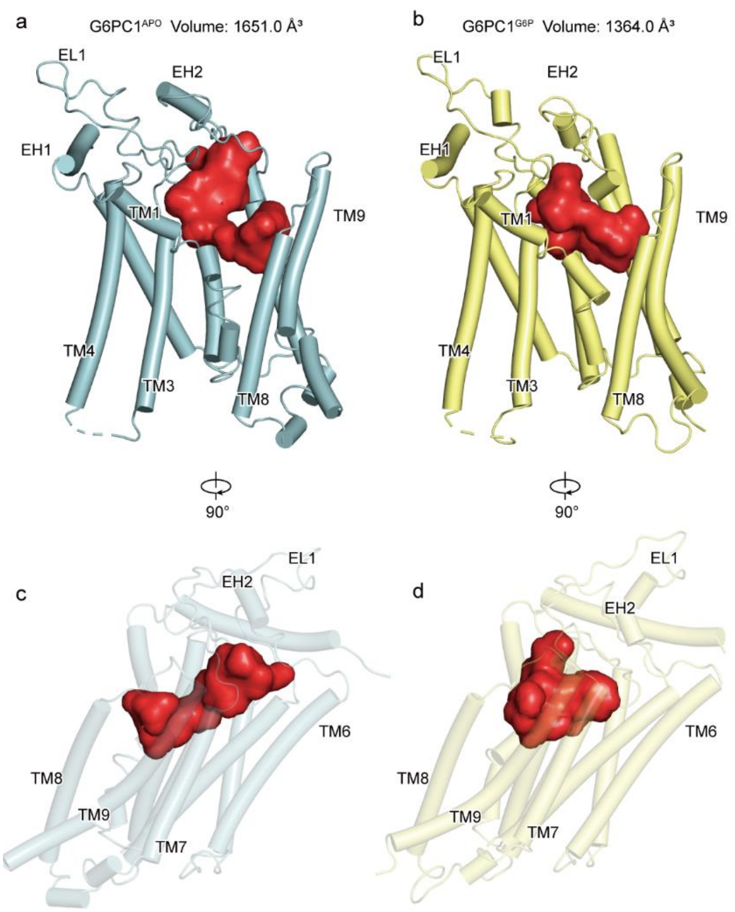

Supplementary Fig. S9 Volume statistics of substrate binding pockets in G6PC1^{APO} and G6PC1^{G6P}

a,c The volume of substrate binding pockets in G6PC1^{APO} from frontal view (a) and side view (c). **b,d** The volume of substrate binding pockets in G6PC1^{G6P} from frontal view (b) and side view (d). Volumes are calculated by PyVOL in PyMOL. Minimum radius is set to 1.4, and maximum radius is set to 5.0.

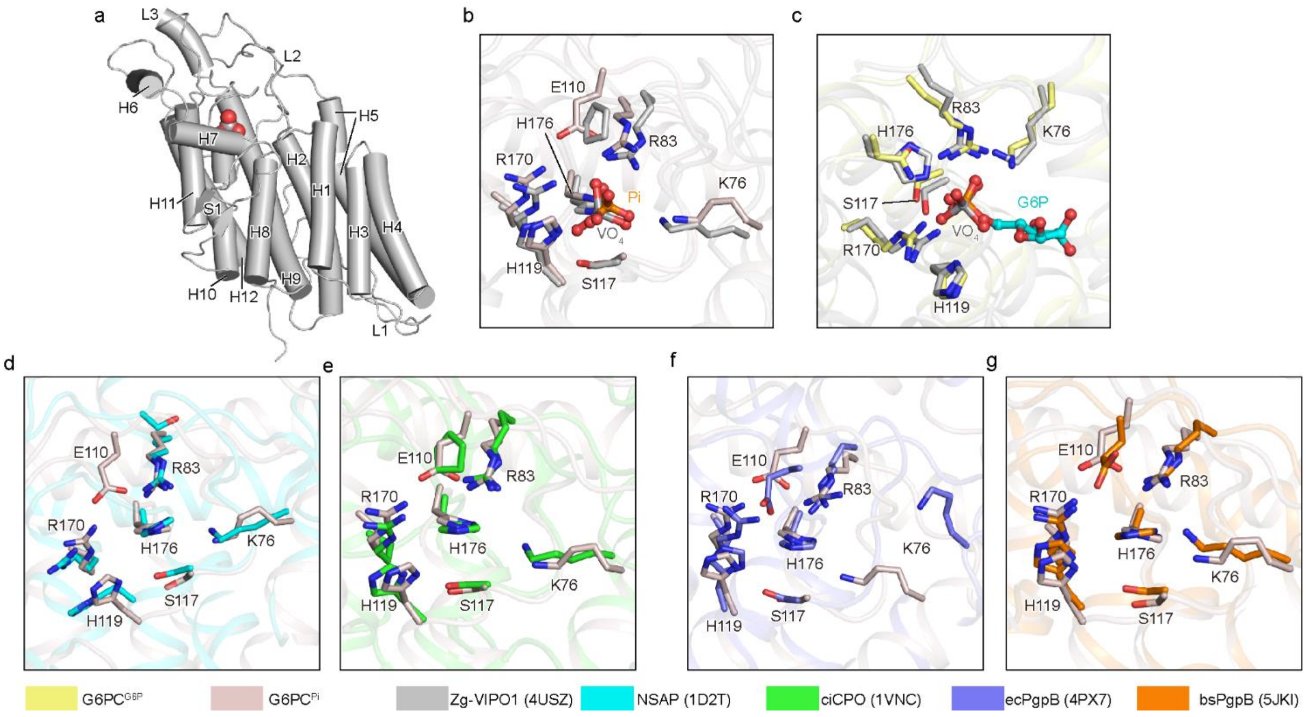

Supplementary Fig. S10 Structural alignment between G6PC1 and several members of PAP2 family.

a Overall structure of Zg-VIPO1 (PDB: 4USZ). The helices are shown as cylinders. The vanadate (VO_4^{3-}) is shown as spheres. The oxygens of VO_4^{3-} are colored red. **b–c** Comparison of the interactions involved in the phosphate binding pocket between Zg-VIPO1 and G6PC1^{Pi} (lightpink) or G6PC1^{G6P} (paleyellow). Interactive residues are shown as sticks, and both G6P and Pi are depicted as ball-and-stick models. **d–g** Alignment of the charge-relay system of G6PC1^{Pi} with other members in PAP2 family. The soluble members are shown in (d–e), and the transmembrane members are exhibited in (f–g). The variant members are colored differently, with corresponding labels shown below.

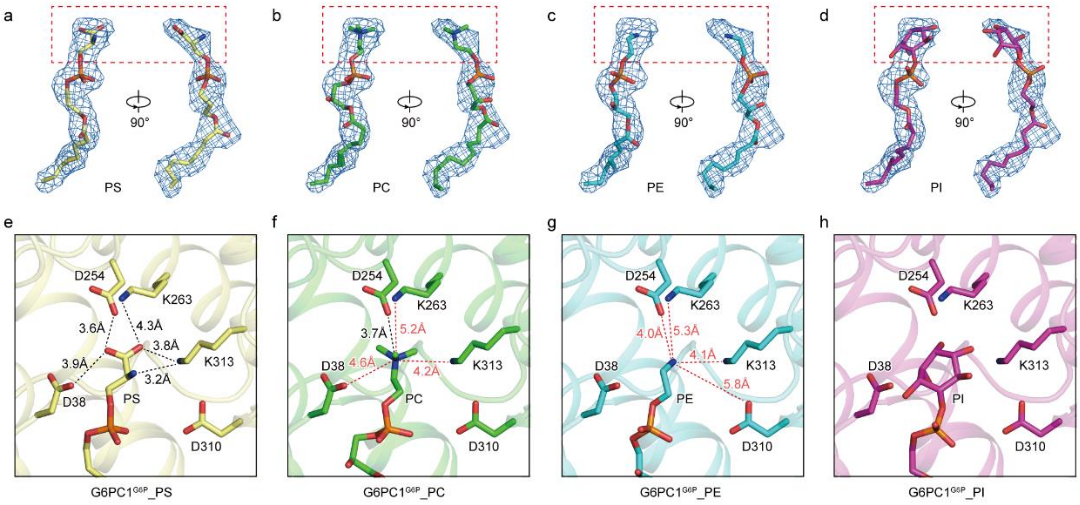

Supplementary Fig. S11 Structural alignment between G6PC1 and several members of PAP2 family.

a-d Matches between different phospholipid molecules and the observed Cryo-EM map. PS, PC, PE and PI are shown in a, b, c and d, respectively. **e-h** The putative interactions between G6PC1 and different phospholipids, including PS (e), PC (f), PE (g) and PI (h). The distances are demonstrated.

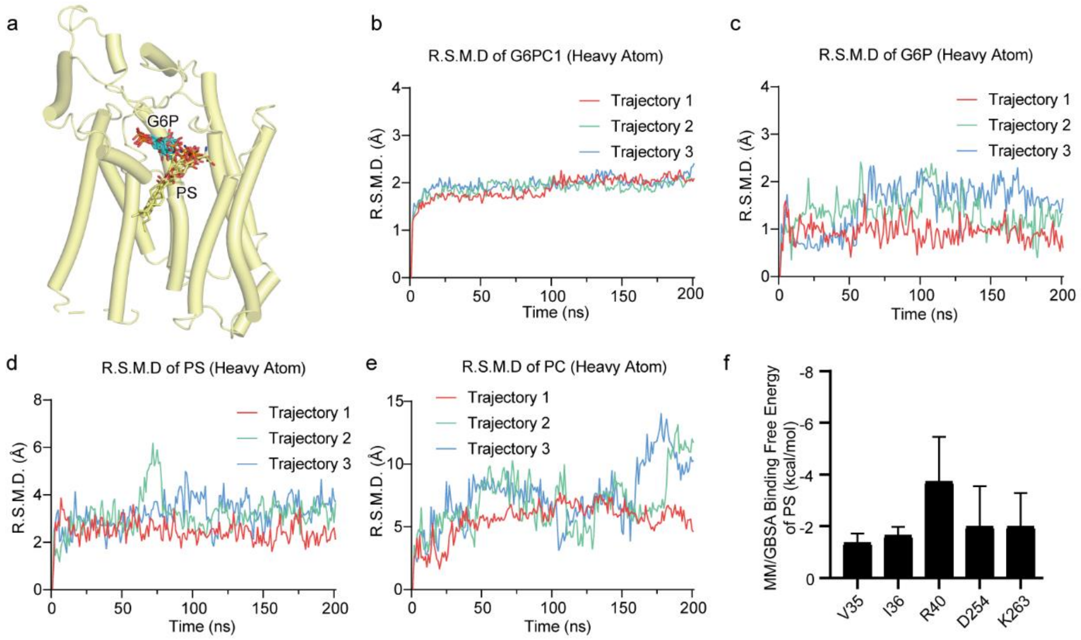

Supplementary Fig. S12 The results of MD stimulations about lipid binding.

a The alignments of binding positions for PS and G6P with G6PC1 in eight simulation trajectories. The structure of G6PC1 is shown as cylinder, and the PS and G6P are depicted as yellow sticks and cyan sticks, respectively. **c-e** The protein backbone (b), G6P (c), PS (d) or PC (e) RMSD plots for each replicate of the simulations. **f** PS binding free energy contribution by key residues in its binding pocket. Data are the mean ± S.D.; n = 3 experimental replicates.

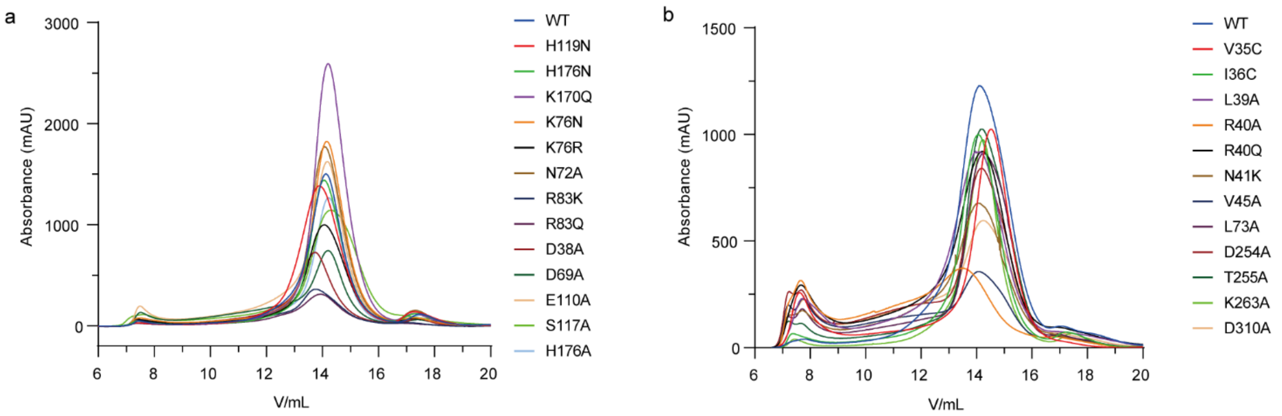

Supplementary Fig. S13 FSEC profiles of mutations.

The mutations within G6P binding pocket are shown in a panel, and the mutations within PS binding pocket are demonstrated in b panel.

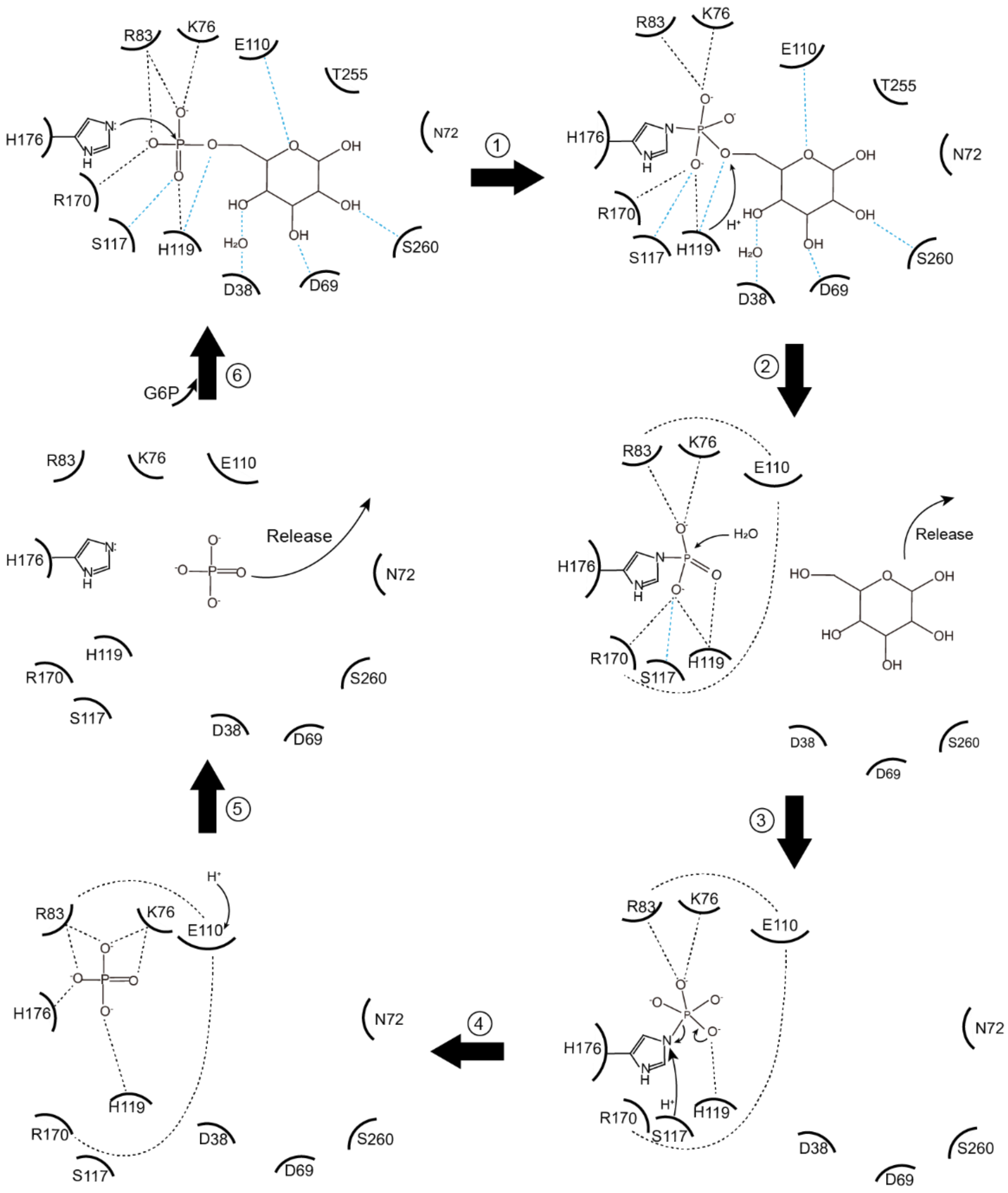

Supplementary Fig. S14 Schematic of catalysis in G6PC1.

Schematic indicates the detailed catalytic process of G6PC1. The interactions are shown as dashed lines. Hydrogen bonds and electrostatic interactions are colored teal and black, respectively. Residues are depicted as black curved lines. The lone pair electrons are shown as black points.

Supplementary Table S1 Cryo-EM data collection, refinement and validation statistics.

	G6PC1^{APO} (EMD-61811) (PDB 9JTL)	G6PC1^{G6P} (EMD-61812) (PDB 9JTM)	G6PC1^{Pi} (EMD-61813) (PDB 9JTN)	G6PC1^{F6P} (EMD-61814) (PDB 9JTO)
**Data collection and processing**				
Magnification	×105,000	×105,000	×105,000	×105,000
Voltage (kV)	300	300	300	300
Electron exposure ($e^{-}/Å^{2}$)	60	60	60	60
Defocus range (μm)	−1.0 – −2.0	−1.0 – −2.0	−1.0 – −2.0	−1.0 – −2.0
Pixel size (Å)	0.85	0.85	0.85	0.85
Symmetry imposed	C1	C1	C1	C1
Initial particle images (no.)	1,261,740	3,256,715	2,405,043	2,804,299
Final particle images (no.)	28,471	56,299	49,677	55,196
Map resolution (Å)	3.4	2.9	3.1	3.3
FSC threshold	0.143	0.143	0.143	0.143
**Refinement**				
Model resolution (Å)	3.7	3.3	3.3	3.6
FSC threshold	0.5	0.5	0.5	0.5
Map sharpening *B* factor ($Å^{2}$)	-106.3	-102.8	-90.8	-140.6
Model composition				
Non-hydrogen atoms	2803	2820	2813	2817
Protein residues	346	346	345	346
Ligands	1	2	2	2
B factors ($Å^{2}$)				
Protein	52.40	83.72	49.38	51.50
Ligand	20.00	78.33	23.92	46.13
R.m.s. deviations				
Bond lengths (Å)	0.003	0.003	0.003	0.003
Bond angles (°)	0.631	0.626	0.639	0.565
Validation				
MolProbity score	1.61	1.59	1.22	1.58
Clashscore	6.44	3.92	2.15	4.82
Poor rotamers (%)	0.00	0.33	0.00	0.33
Ramachandran plot				
Favored (%)	96.20	93.86	96.48	95.32
Allowed (%)	3.80	6.14	3.52	4.68
Disallowed (%)	0.00	0.00	0.00	0.00
